# T-tubule remodelling disturbs localized β_2_-adrenergic signalling in rat ventricular myocytes during the progression of heart failure

**DOI:** 10.1093/cvr/cvx074

**Published:** 2017-05-13

**Authors:** Sophie Schobesberger, Peter Wright, Sergiy Tokar, Anamika Bhargava, Catherine Mansfield, Alexey V. Glukhov, Claire Poulet, Andrey Buzuk, Aron Monszpart, Markus Sikkel, Sian E. Harding, Viacheslav O. Nikolaev, Alexander R. Lyon, Julia Gorelik

**Affiliations:** 1 Department of Cardiac Medicine, National Heart and Lung Institute, Imperial College, Du Cane Road, London W12 0NN, UK;; 2 Institute of Experimental Cardiovascular Research, University Medical Center Hamburg-Eppendorf, Martinistraße, Hamburg D-20246, Germany;; 3 Department of Biotechnology, Indian Institute of Technology Hyderabad, Ordnance Factory Estate, Yeddumailaram, 502205 Telangana, India;; 4 Department of Computer Science, University College London, Gower Street, London WC1E 6BT, UK;; 5 NIHR Cardiovascular Biomedical Research Unit, Department of Cardiology, Royal Brompton Hospital, Sydney Street, London SW3 6NP, UK

**Keywords:** Heart failure, Hypertrophy, Remodelling, β-Adrenoreceptor signalling, T-tubules, Scanning ion conductance microscopy

## Abstract

**Aims:**

Cardiomyocyte β_2_-adrenergic receptor (β_2_AR) cyclic adenosine monophosphate (cAMP) signalling is regulated by the receptors’ subcellular location within transverse tubules (T-tubules), via interaction with structural and regulatory proteins, which form a signalosome. In chronic heart failure (HF), β_2_ARs redistribute from T-tubules to the cell surface, which disrupts functional signalosomes and leads to diffuse cAMP signalling. However, the functional consequences of structural changes upon β_2_AR-cAMP signalling during progression from hypertrophy to advanced HF are unknown.

**Methods and results:**

Rat left ventricular myocytes were isolated at 4-, 8-, and 16-week post-myocardial infarction (MI), β_2_ARs were stimulated either via whole-cell perfusion or locally through the nanopipette of the scanning ion conductance microscope. cAMP release was measured via a Förster Resonance Energy Transfer-based sensor Epac2-camps. Confocal imaging of di-8-ANNEPS-stained cells and immunoblotting were used to determine structural alterations. At 4-week post-MI, T-tubule regularity, density and junctophilin-2 (JPH2) expression were significantly decreased. The amplitude of local β_2_AR-mediated cAMP in T-tubules was reduced and cAMP diffused throughout the cytosol instead of being locally confined. This was accompanied by partial caveolin-3 (Cav-3) dissociation from the membrane. At 8-week post-MI, the β_2_AR-mediated cAMP response was observed at the T-tubules and the sarcolemma (crest). Finally, at 16-week post-MI, the whole cell β_2_AR-mediated cAMP signal was depressed due to adenylate cyclase dysfunction, while overall Cav-3 levels were significantly increased and a substantial portion of Cav-3 dissociated into the cytosol. Overexpression of JPH2 in failing cells *in vitro* or AAV9.SERCA2a gene therapy *in vivo* did not improve β_2_AR-mediated signal compartmentation or reduce cAMP diffusion.

**Conclusion:**

Although changes in T-tubule structure and β_2_AR-mediated cAMP signalling are significant even at 4-week post-MI, progression to the HF phenotype is not linear. At 8-week post-MI the loss of β_2_AR-mediated cAMP is temporarily reversed. Complete disorganization of β_2_AR-mediated cAMP signalling due to changes in functional receptor localization and cellular structure occurs at 16-week post-MI.

## 1. Introduction

Heart failure (HF) is a complex clinical syndrome arising from myocardial injury or dysfunction. Together with other cardiovascular complications, HF accounts up to 40% of all deaths worldwide.^1^ HF does not occur instantaneously but develops over time due to mechanical and hormonal stresses and adverse cardiac remodelling, as shown in multiple human studies and recapitulated in animal models.^2^ The stages of disease progression may manifest themselves, at first, as compensatory hypertrophy then as decompensated hypertrophy and finally as HF. When HF develops, ventricular myocytes undergo a plethora of functional and structural changes.^3^^,^^4^ During the initial phase of HF progression, the sympathetic nervous system compensates for the diminished cardiac output through an increase in catecholaminergic stimuli^3^ and cardiomyocytes experience hypertrophic growth.^5^ This results in transiently advantageous changes in the β-adrenergic receptor (β-AR)- and cyclic adenosine monophosphate (cAMP)-dependent inotropic response of cardiomyocytes^4^ but transforms into a maladaptive decrease of myocardial responsiveness at more advanced stages of HF.^6^^,^^7^

The β_1_- and β_2_ARs constitute the predominant receptor pathways for the sympathetic control of myocardial function. Diverse mechanisms have evolved to translate signalling via these two molecules into differential effects on physiology. β_1_ARs but not β_2_ARs stimulate the cAMP-dependent protein kinase (PKA)-mediated phosphorylation of phospholamban and cardiac contractile proteins,^8^ induce hypertrophy upon moderate overexpression,^9^^,^^10^ and promote cardiomyocyte apoptosis.^8^^,^^11^ In mouse ventricular myocytes, we have shown that, upon local receptor stimulation, β_1_-AR-mediated cAMP signalling propagated throughout the entire cell, whereas β_2_AR-cAMP responses were locally confined.^12^ In HF, secondary to changes in cardiomyocyte structure, the β_2_AR-cAMP response was no longer exclusively detectable in the transverse tubules (T-tubules) but also appeared in the crests, dome shaped, interspersing plasma membrane areas between T-tubule openings.^13^ This results in uncoupling of β_2_ARs from the localized pools of PKA that are responsible for the compartmentation of the β_2_AR-cAMP signalling.^14–16^ Thus, in failing cells, activation of β_2_ARs leads to cell-wide cAMP signal propagation patterns similar to the patterns observed for β_1_ARs, which may account for the loss of its normally cardioprotective properties and its contribution to the HF phenotype.^17^ The transverse axial tubule (TAT) system, which includes the T-tubules as well as longitudinally directed elements, plays an important role in signal transduction and cell homeostasis.^18^ It develops gradually in postnatal cardiomyocytes in a process, which is associated to the structural regulation and modification by multiple structural proteins, including among many others junctophilin-2 (JPH2) and the T-tubule as well as JPH2 associated Caveolin 3 (Cav-3).^19^ Although JPH2 appears to anchor the SR close to the T-tubule membranes,^20^ Cav-3 has been associated to caveolae, nanometer sized, bubble-like invaginations of the cell membrane, which may be the precursors of T-tubules.^21^ Cav-3 has also been shown to play a prominent role in cell signal regulation^22^ including the β_2_AR signalling pathway.^23^ Alterations in T-tubule associated structural proteins and disruption of the TAT system have been shown to be concurrent with HF in animal models and human cardiomyocytes.^24^^–^^27^ As HF is a progressive disease, the time dependent changes in cardiomyocytes regarding β_2_AR-cAMP signalling and cell structure during the progression of left ventricular (LV) hypertrophy to advanced HF need further investigation.

Here, we hypothesized that disruption of T-tubular microdomains during the progression of LV hypertrophy to advanced HF, may have an impact on β_2_ARs-cAMP response location thus promoting diffuse pathological signalling. We used scanning ion conductance microscopy (SICM), Förster Resonance Energy Transfer (FRET) and confocal imaging to visualize cell surface topography and T-tubule network and to investigate the compartmentalization of β_2_AR-cAMP signalling in rat LV myocytes isolated at 4-, 8-, and 16-week post-myocardial infarction (MI). In cardiomyocytes transduced with the Epac2-camps cAMP-FRET sensor, β_2_ARs were stimulated either via whole-cell perfusion or locally (in or outside of the T-tubule) via a SICM nanopipette. In addition, cAMP diffusion was investigated in cardiomyocytes isolated from 16-week post-MI rats, which underwent sarcoplasmic reticulum Ca^2+^ pump 2a (SERCA2a) gene therapy. In tandem we investigated the total protein levels of the T-tubule associated proteins JPH2 and Cav-3 as well as Cav-3 levels in the membrane and cytosol fractions of cells.

## 2. Methods

Detailed methods and further information are provided in the Supplementary material online.

### 2.1 Animals

All procedures were carried out according to the standards for the care and use of animal subjects determined by the UK Home Office (ASPA1986 Amendments Regulations 2012) incorporating the EU directive *2010/63/EU*. The Animal Welfare and Ethical Review Body Committee of Imperial College London approved all protocols.

### 2.2 MI model

**Figure 1:**
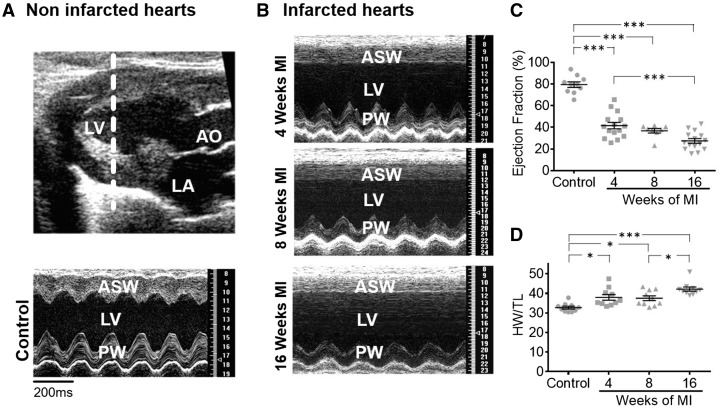
Rat heart size increases while heart function declines progressively post-MI *(A)* Echocardiography data; Top panel: Echocardiography image indicating the plane (white punctuated line) in the LV from which echocardiography data represented in the bottom panel was collected, over time. LA marks the location of the left atrium and AO marks the aorta. Bottom panel: echocardiography data showing the ASW and the PW function of the LV. *(B)* Echocardiography data at different time points post-MI; *(C)* Ejection fraction at different time points post-MI, sample numbers in control followed by chronological order of MI samples: *n* = 10, *n* = 15, *n* = 8, *n* = 15; *(D)* Heart weight corrected to TL at different time points post-MI, each time point *n* = 10; Control rats were from an even spread of time points 4 at 4-week, 4 at 8-week, and 3 at 16-week post-MI; *N*, number of animals, **P < 0.001, ***P < 0.0001 as determined with one-way ANOVA followed by Bonferroni *post hoc* correction.

Myocardial infarction (MI) was induced by ligation of the left anterior descending coronary artery of male, adult Sprague-Dawley rats as described previously in^24^ and in the supplementary detailed methods section. Animals were only used for operation once they had reached a body weight of 250 g. Echocardiography measurements in M-mode were performed (*Figure 1A*) on some of the animals. Before animals were sacrificed by cervical dislocation they were exposed to 5% isoflurane until they lost their righting reflex. Body weight, heart weight, tibia length (TL) and anteroseptal as well as posterior wall (PW) thickness were determined from the sacrificed animals. Cardiomyocytes were isolated from the LV of age matched control hearts and hearts 4-, 8-, and 16-week post-MI.

### 2.3 Scanning of cardiomyocyte surface, TAT structure evaluation and cell size determination

Cardiomyocyte topography scans were obtained using SICM with nanopipettes of ∼100 MΩ resistance, as described previously.^28^ The Z-groove index was calculated to quantify changes in surface structures of control and MI cells, as described previously in.^29^

Cardiomyocytes were stained with the lipophilic dye Di-8-ANEPPS and imaged using an inverted confocal microscope (Zeiss LSM-780) to measure T-tubule regularity, density and the amount of longitudinal elements as well as single cardiomyocyte lengths, heights, widths and overall cell volume (see supplementary material online, *Figure S2*). Three-dimensional representations of the T-tubule network were generated from confocal images (see Supplementary material online, *Movies*). For illustration of how the aforementioned parameters were determined see supplementary material online, *FigureS8* and *S9*.

### 2.4 JPH2 and caveolin-3 protein level quantification and cell fractionation

Relative amounts of JPH2 and caveolin-3 (Cav-3) were measured by densitometry of protein bands following Western blotting with specific antibodies (JPH-2 goat polyclonal, Santa Cruz, sc-51313; Cav-3 monoclonal mouse, Santa Cruz, sc-5310; and normalization to glycerinaldehyd-3-phosphat-dehydrogenase (GAPDH) (mouse monoclonal, BioTrend Chemikalien GmbH, no. 5G4-6C5) and calsequestrin (CSQ) (rabbit polyclonal, Thermo Scientific, PA1-913) levels. For this, cells from three animals for each condition were used and all blots were performed in three replicates. Additionally, Cav-3 levels in membrane and cytosolic fractions of control cells and cardiomyocytes at 4- and 16-week post-MI were determined. In order to investigate Cav-3 dissociation from the membrane into the cytosol, we took advantage of the protein not being dissolvable in Triton, but sodium dodecyl sulphate and performed cell fractionation as is described in detail in the supplementary material online.

### 2.5 Measurement of ß2AR-dependent cAMP signalling after whole-cell stimulation

The amount of cAMP produced following activation of β_2_AR was determined with a FRET-based method. Cardiomyocytes were transduced with adenovirus encoding Epac2-camps construct for 48 hours as described previously in.^13^ Then the cells were pre-incubated with β_1_-AR blocker CGP20712A (CGP, 100 nmol/L) for 5 min before perfusing with nonspecific ß-AR agonist isoproterenol (ISO, 100 nmol/L) to measure β_2_AR-dependent cAMP production. At the end of the experiment, maximal cAMP production was measured by activation of adenylate cyclase (AC) using the forskolin analogue NKH477 (NKH, 5µmol/L).

### 2.6 Measurement of ß2-AR-dependent cAMP signalling after local stimulation

Control cells and cells post-MI were transduced as above. Topographical images were obtained by SICM. CGP20712A (100 nmol/L) was added to the cell bath at least 5 min before β_2_ARs were stimulated locally via the SICM nanopipette by pressure application of the agonist into either a T-tubule opening or onto a crest area with pipette solution containing ISO (10 µmol/L) and CGP20712A (10 µmol/L) as described previously^13^. Additionally, cells 4-week post MI were pre-treated with pertussis toxin (PTX, 1.5 µg/ml) for 3 h before local stimulation. β_2_AR-dependent cAMP diffusion distance was determined for control cells and cells isolated from animals post-MI and from animals which underwent SERCA2a gene therapy as described previously in.^30^

### 2.7 Statistical analysis

A two-tailed *t*-test was used for the comparison between independent groups of normally distributed data as determined by Kolmogorov-Smirnov testing. One-way analysis of variance (ANOVA) followed by Bonferroni correction *post hoc* testing was used for simple two-group comparison. Datasets of a hierarchical nature, i.e. data for which single cardiomyocytes were assigned to their respective animals and sub-grouped accordingly, were compared with a mixed ANOVA followed by Wald χ^2^-test. All data are presented as scatter/dot plots with means and standard errors.

## 3. Results

### 3.1 HF phenotype progression

**Figure 2:**
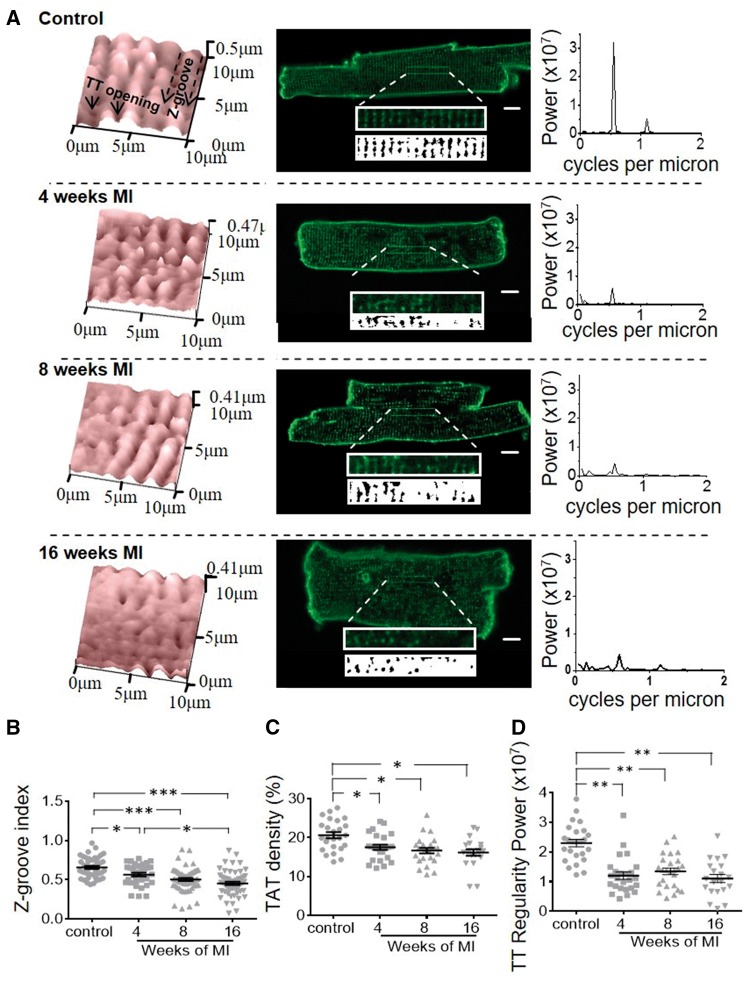
Cardiomyocyte surface structures, T-tubule density and regularity decrease and remain decreased from the earliest time point post-MI. *(A)* Structural features of cardiomyocytes at various time points; Left panels: representative 10 × 10µm cell topography scans of cardiomyocytes isolated from control rat hearts and at different time points post-MI (exemplary Z-grooves and T-tubule openings are highlighted with black arrows). Middle panels: representative confocal images taken from control and failing cardiomyocytes at different time points post MI. Scale bars = 10 µm. Top insets in confocal images: zoom of 40 × 5 µm area of the confocal image; Bottom insets: binarized image represented in the corresponding top inset, used to determine T-tubule regularity; Right panels: power peaks of T-tubule regularity calculated from confocal images via 1D Fourier Transformation; a single, high peak indicates regular T-tubule distribution at the corresponding distance. *(B)* Z-groove index, an indicator of surface structural integrity. Sample numbers in control followed by chronological order of MI samples: *N/n* = 9/44, *N/n* = 7/32, *N/n* = 11/60, *N/n* = 9/56; *(C)* Density of T-tubules calculated from binarized images (TAT density). Sample numbers in control followed by chronological order of MI samples: *N/n* = 5/25, *N/n* = 5/25, *N/n* = 5/25, *N/n* = 4/20 *(D)* Power of the major T-tubule regularity peak calculated from binarized images via 1D Fourier Transformation (regularity power). Sample numbers in control followed by chronological order of MI samples: *N/n* = 5/25, *N/n* = 5/25, *N/n* = 5/25, *N/n* = 4/20; *N*, number of animals, *n*, number of cells, *P < 0.05, **P < 0.001, ***P < 0.0001 as calculated with mixed ANOVA followed by Wald χ^2^-test.

After coronary ligation, the anteroseptal wall (ASW) of the LV becomes scarred and akinetic (*Figure 1A*). In addition, there was progressive dysfunction and hypokinesis of the PW, which was clearly seen at 16-week post-MI (*Figure 1B*). The LV ejection fraction declined progressively over time, with the greatest reduction occurring between 8- and 16-week post-MI (*Figure 1C*). Eccentric, single cell hypertrophy subsequently developed as early as 4-week post-MI to compensate for reduced muscle function (see supplementary material online, *FigureS2B*) and an overall increase in cardiomyocyte volume was detectable at 8- and 16-week post-MI (see supplementary material online, *FigureS2C*,*D)*. By 4-week post-MI, there was a marked increase of heart weight to TL ratio (by ∼16% compared with controls, *P* < 0.05) with a gradual further increase over time (*Figure 1D*). Hence, consistent with progressive adverse remodelling observed in humans,^27^ MI rats also experience progressive cardiac remodelling.

### 3.2 TAT network remodelling

**Figure 3:**
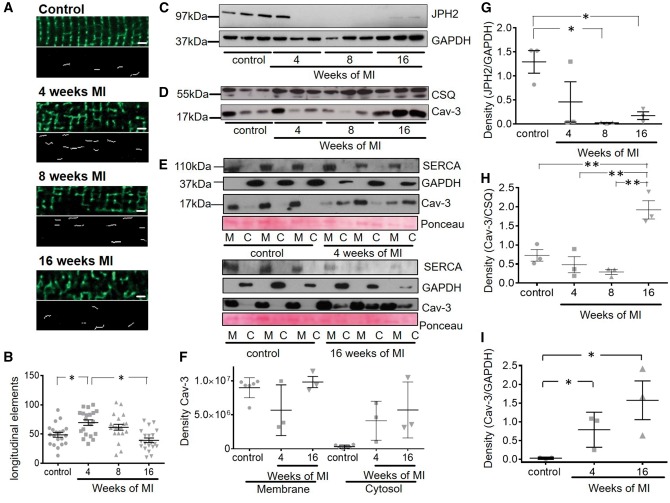
Transiently increased occurrence of longitudinal elements in the TAT system is accompanied by a persistent decrease in JPH2 post-MI and dissociation of Cav-3 into the cytosol. The occurrence of longitudinal elements of the TAT system transiently rises while JPH2 levels decrease and remain decreased from the earliest time point post-MI and Cav-3 levels increase and dissociate from the membrane 16-week post-MI. *(A)* Top panels: representative 40 × 10 µm frames of the confocal images of cardiomyocytes at different time points (Scale bar equals 2 μm); Bottom panels: axial elements extracted mathematically from frames above as explained in ‘Methods’ section; *(B)* Number of longitudinal tubules; *P < 0.05 as calculated with mixed ANOVA followed by Wald χ^2^-test. Sample number *N/n* = 4/20 each; *(C)* Representative immunoblots for JPH2 and *(D)* of Cav-3 as well as the house keeping gene products GAPDH and CSQ, each sample was analysed in triplicates from 3 different animals (control = age matched animals); *(E)* Representative immunoblots showing dissociation of Cav-3 protein from membrane to cytosol at 4- and 16-week post-MI. SERCA2a was used as membrane fraction marker and GAPDH as cytosolic fraction marker. Ponceau staining was performed as overall loading control. *(F)* and *(G)* Density of respective JPH2 bands in (C) normalized to corresponding GAPDH bands, *n* = 3 each, and of Cav-3 bands in (D) normalized to CSQ bands. *(H)* Density of Cav-3 in cytosolic fractions of cardiomyocytes 4- and 16-week post-MI normalized to GAPDH in (E). Sample number *N* = 3 each, *N*, number of animals; *n*, number of cells. *P < 0.05 as determined with one-way ANOVA followed by Bonferroni *post hoc* correction.

In healthy cardiomyocytes, the TAT network spans the entire cytoplasm and consists of periodically spaced T-tubules and irregular longitudinal elements that connect the T-tubules (*Figure 2A*, middle panels).^31^ On the cardiomyocyte surface, T-tubule openings could be clearly visualized by SICM, which allowed a detailed characterization of topographical structures, with clear distinction of T-tubule openings and crests between Z-grooves (*Figure 2A*, left panels). Profile measurements of topographical images showed that in adult, healthy ventricular myocytes, spacing between Z-grooves is ≈2 μm, corresponding to the sarcomere length.^29^ HF resulted in significant remodelling in both TAT and topography structures (*Figure 2A*). Selected area of confocal images of cells stained with membrane dye Di-8-ANEPPS were analysed for the regularity of T-tubule elements by Fast Fourier transformation. Consistent with other studies^25^ our calculations of T-tubule regularity indicate that they were interspaced highly regularly at ∼2 µm intervals in healthy cardiomyocytes. In addition, the density of the network was measured on these images. T-tubule regularity dropped drastically by ∼47% (*P* = 0.004) (*Figure 2D*) together with a smaller, but significant reduction in TAT density by ∼11% (*P* = 0.016) as early as 4-week post-MI, with no further changes at later time points (*Figure 2C*). Along with TAT remodelling, cardiomyocytes from failing rats displayed alterations in their surface structure,^29^ which were reflected by a reduced Z-groove index. At 4-week post-MI, a small decrease in the Z-groove index by ∼9% (*P* = 0.0002) was detected (*Figure 2B*). In contrast to the TAT remodelling, the reduction of Z-grooves progressed with time, with a further 9% decrease at 8- and 16-week post-MI (*Figure 2B*). Interestingly, in contrast to the reduction in T-tubule density and regularity, the number of longitudinal elements of the TAT network increased by ∼41%(*P* = 0.040) at 4 weeks (*Figure 3A,B*) and remained elevated until 8 weeks before declining in line with the overall T-tubule organization at 16-week post-MI.

The membrane bridging protein JPH2 has been implicated in determining T-tubule orientation and formation.^20^ We therefore examined whether the abundance of JPH2 protein changes along with TAT remodelling. Western blot results demonstrated that JPH2 expression was already dramatically reduced as early as 4-week post-MI in two out of three samples, although there was variability in animals at that stage and hence the difference was not statistically significant. The differences had become significant by 8-week post-MI (*P* < 0.05) and remained low throughout at the end stage of HF (*Figure 3C*). Cav-3, another structural protein associated with JPH2 and T-tubular structure, started partially dissociating into the cytosol (∼44% of the overall protein in control, when adding up membrane and cytosol amounts) at 4-week post-MI (*P* < 0.05) (*Figure 3E,F,I*). Then, at 16-week post-MI, it was significantly increased about 2.6-fold in comparison to control (*P* < 0.05) (*Figure 3D*). This increase was accompanied by a significant (*P* < 0.05) and even stronger dissociation of Cav-3 (∼61% of the overall protein in control, when adding up membrane and cytosol amounts) from the membranes into the cytosol of failing cardiomyocytes (*Figure 3E,F,H,I*). Whole cell Cav-3 and fractionized Cav-3 level measurements were conducted in different samples from different animals, hence normalized values differ between blots. Fractionized Cav-3 levels were normalized against GAPDH as this house keeping gene is simultaneously a cytosolic marker, while overall Cav-3 levels were normalized against CSQ.

### 3.3 **β**_2_AR-dependent cAMP response after whole-cell activation

**Figure 4:**
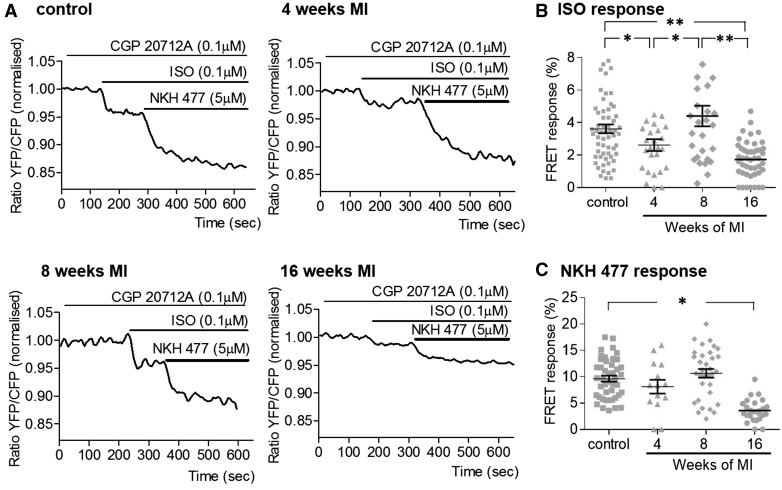
The intracellular cAMP response to whole-cell β_2_AR stimulation undergoes a temporary decline followed by an increase before its depression at the end stage of HF. *(A)* Representative traces of the whole-cell FRET following β_2_AR-specific stimulation with ISO in the presence of selective β_1_AR antagonist CGP20712A; YFP/CFP ratio is inversely related to the cAMP level; AC agonist NKH477 was later added to elicit maximal cAMP production; *(B)* Whole-cell FRET response to the selective β_2_AR stimulation with ISO at different time points after MI. Sample numbers in control followed by chronological order of MI samples: *N/n* = 9/61, *N/n* = 5/26, *N/n* = 7/32, *N/n* = 9/41; *(C)* Whole-cell FRET response to NKH477 showing maximal cAMP production. *N*, number of animals; *n*, number of cells. Sample numbers in control followed by chronological order of MI samples: *N/n* = 7/38, *N/n* = 4/14, *N/n* = 7/32, *N/n* = 6/26. *P < 0.05, **P < 0.001 as calculated with mixed ANOVA followed by Wald χ^2^-test.

Representative FRET ratio traces recorded in control and cells at 4-, 8-, and 16-week post-MI during β_2_AR stimulation are shown in *Figure 4A*. We found that at 4-week post-MI, the whole-cell β_2_ARs-cAMP response dropped slightly, but significantly by ∼30% (*P* = 0.024); however at 8 weeks, we saw the level of response recover to the level observed in control cells (*Figure 4B*). At the same time, maximum AC activity did not change significantly at the first two time points (*Figure 4C*). At 16-week post-MI however we observed a concurrent, significant decrease in both β_2_AR-dependent cAMP levels by ∼48%, (*P* = 0.011) and the overall cAMP level produced after AC stimulation by ∼63% (*P* = 0.014), as compared with control cells (*Figure 4B,C*).

### 3.4 **β**_2_AR localization and cAMP signal compartmentation

**Figure 5:**
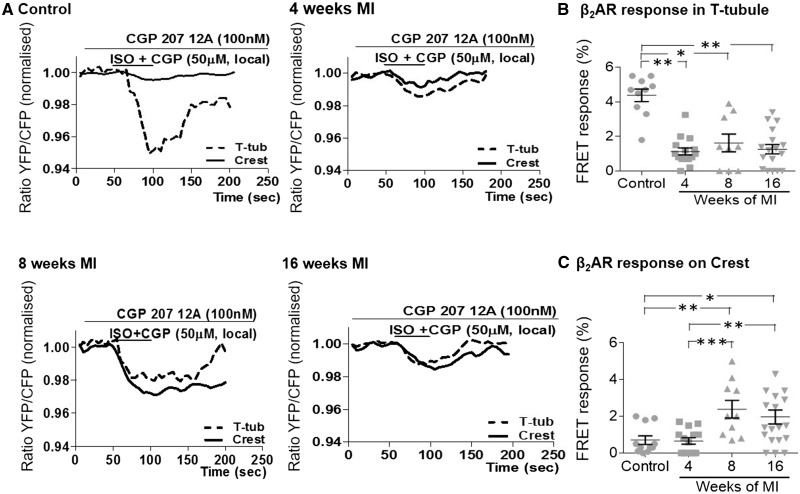
The β_2_AR-dependent cAMP response to local stimulation, dissipates from T-tubules early post-MI to crests at a later time point post-MI. *(A)* Representative curves of FRET response after local β_2_AR stimulation either in T-tubule openings (dotted line) or on crest areas (black line). Local β_2_ARs-dependent cAMP FRET response after agonist application *(B)* into T-tubules, sample numbers in control followed by chronological order of MI samples: *N/n* = 5/10, *N/n* = 6/15, *N/n* = 4/9, *N/n* = 5/17, or *(C)* onto crest, sample numbers in control followed by chronological order of MI samples: *N/n* = 5/11, *N/n* = 5/14, *N/n* = 4/10, *N/n* = 6/19. *N*, number of animals; *n*, number of cells. *P < 0.05, **P < 0.001, **P < 0.0001 as calculated with mixed ANOVA followed by Wald *χ*^2^-test.

To study compartmentation of the cAMP response in T-tubules, we stimulated β_2_ARs locally via the SICM nanopipette. Typical traces of FRET ratios recorded following local β_2_AR stimulation on the T-tubules or on the crests are presented in *Figure 5A*. As we reported previously in,^13^ we observed that in control cardiomyocytes, the β_2_AR-dependent cAMP response is five-fold greater in the T-tubule openings than at the crests (*Figure 5A,B,C*), whereas in cardiomyocytes isolated from rat hearts at end stage HF (16-week post-MI), the β_2_AR-cAMP response was found to be equally present in T-tubules and in the crests (*Figure 5A,B,C*). Here we aimed to study when this β_2_AR-cAMP response appearance at the crests occurs first. We found that β_2_AR-dependent cAMP response following stimulation in T-tubules was strongly reduced as early as 4-week post-MI (∼72%, *P* = 0.004; *Figure 5B*), whereas no change was detected following stimulation at crest areas compared with control cells. Moreover, while the β_2_AR-dependent cAMP response at the T-tubules remained low at 8-week post-MI, response to crest stimulation increased significantly (∼300%, *P* = 0.040; *Figure 5C*). There were no further changes in the appearance of β_2_AR-dependent cAMP response at 16-week post-MI (*Figure 5*B,C). Thus, the drop in β_2_AR responsiveness at the T-tubules precedes the appearance of β_2_AR-cAMP signals at the crests.

### 3.5 Diffusion of the **β**_2_AR-dependent cAMP signal

**Figure 6:**
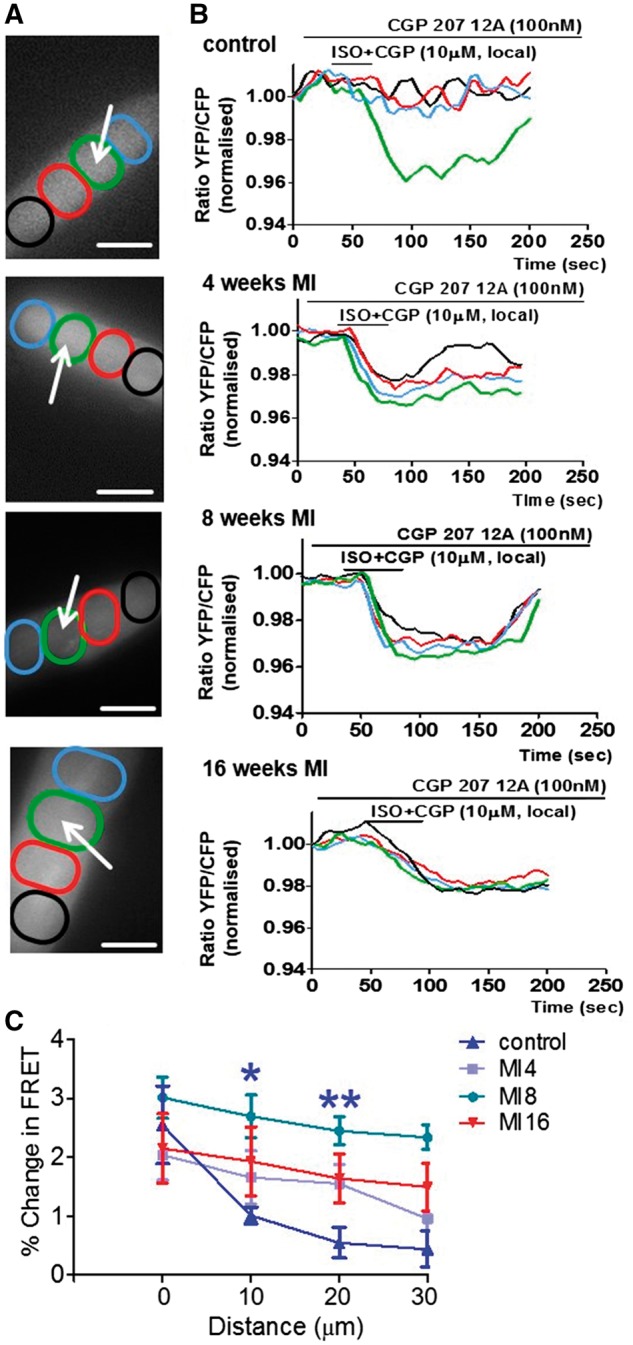
The β_2_AR-dependent cAMP response, which is confined in control cells diffuses throughout the whole cytosol at different time points post-MI. *(A)* images of cardiomyocytes indicate areas of interest (coloured ovals) situated at various distances from the point of stimulation (white arrows) where FRET was measured after local selective stimulation of β_2_ARs in T-Tubules; Scale bars in, 20 µM *(B)* colour-coded curves of FRET response corresponding to the four areas of interest as indicated on the right panels.*(C)* Differences of the relative β_2_AR-cAMP levels at progressive distances from the point of original agonist application in comparison to control cardiomyocytes, N = 5/n = 1 each; *N*, number of animals; *n*, number of cells. ‘*’ indicates a significant difference in cAMP signal level from control, *P < 0.05 as determined by one-way ANOVA followed by Bonferroni *post hoc* correction.

We have previously shown that cells at 16-week post-MI exhibit a β_2_AR-dependent cAMP signal that diffuses throughout the entire cytosol after local stimulation at detubulated areas.^13^ Here we explored the development of this loss of spatial confinement of β_2_AR-dependent cAMP signalling after local stimulation in the T-tubule. We considered a response as local if the signal had dropped by >50% at 30 µm distance from the original site of stimulation. We found that following local β_2_AR stimulation, cAMP diffused throughout the cytosol as early as 4-week post-MI (*Figure 6A,B*), even though β_2_AR—cAMP signals had not relocated yet to the crest at that time point (*Figure 5*). The relative cAMP levels at increasing distance to the original site of stimulation inside T-tubules of cardiomyocytes post infarction were significantly different from control cells, except for control and cells 4-week post-MI at the furthest distance to the original site of application (*Figure 6A,B*). This indicates that cardiomyocytes at 4-week post MI to some degree still have some limited cAMP confinement in the cytosol in comparison to the later stages post-MI.

### 3.6 SERCA2a therapy restores T-tubular localization of **β**_2_ARs but does not confine **β**_2_AR-cAMP response

**Figure 7:**
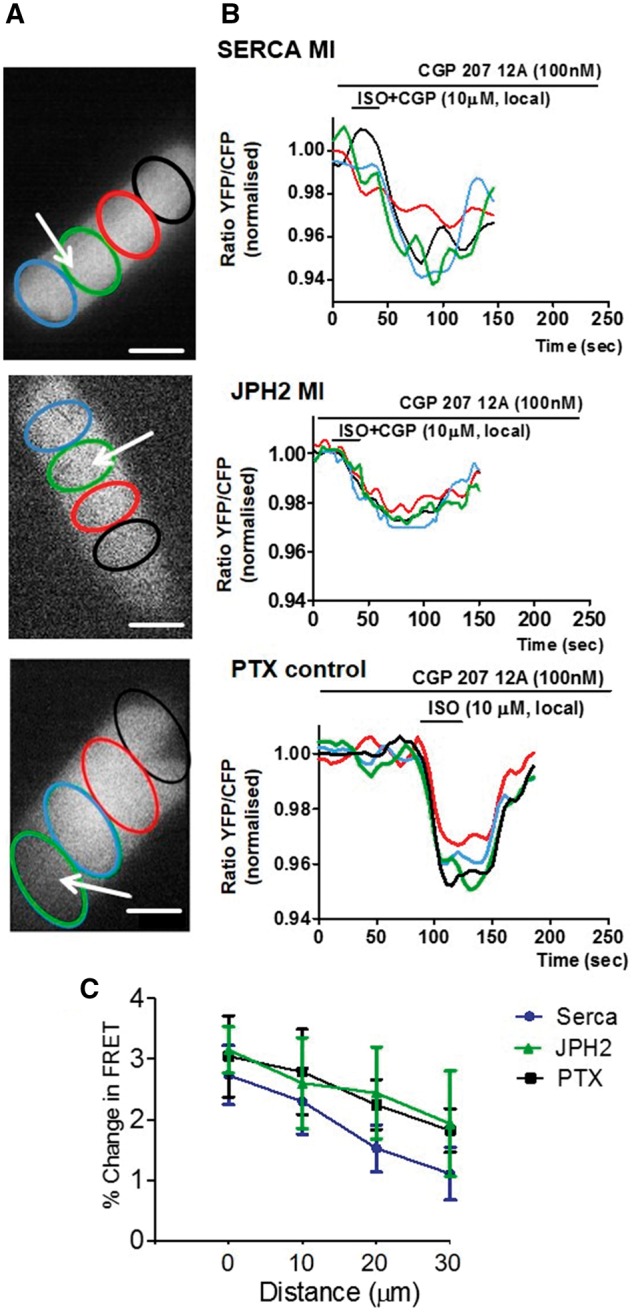
SERCA2a or JPH2 overexpression in post-MI cells do not restore the confinement of the β_2_AR-dependent cAMP response but the loss of confinement can be mimicked by PTX treatment of control cells. *(A)* images of cardiomyocytes indicating areas of interest (coloured ovals) situated at various distances from the point of stimulation (white arrows) where FRET was measured after local selective stimulation of β_2_ARs; Scale bar, 20 µm; *(B)* colour-coded curves of FRET response corresponding to the four areas of interest as indicated on the right panels; from top to bottom: Local FRET in cardiomyocytes isolated from a heart with chronic MI after SERCA2a gene therapy *N/n* = 4/14; Local FRET in cardiomyocytes from a heart with chronic MI overexpressing JPH2 *N/n* = 2/7 (*n* = 7, from two animals); Local FRET in normal cardiomyocytes treated with PTX *N/n* = 3/9. *(C)* Differences of the relative β_2_AR-cAMP levels at progressive distances from the point of original agonist application, average of *N* = 5/*n* = 1 each. *N*, number of animals; *n*, number of cells.

Loss of cAMP confinement during the progression of HF seems to correlate better with loss of TAT system regularity and JPH2 expression than with β_2_AR-cAMP response appearance at the crests. Previously, we have shown amelioration of surface structure organization and TAT network regularity in failing rat ventricular myocytes after gene therapy with SERCA2a expressing adeno-associated virus.^30^ In our previous publication,^30^ we demonstrated that the expression of many structural proteins, including BIN-1 and Tcap, was recovered together with SERCA2a levels and that the contractile function in SERCA2a-treated animals was improved in comparison to untreated animals. Here, we re-examined these cells with regard to their cAMP compartmentation and found that the β_2_AR-dependent cAMP signal is still diffuse as in HF cells (*Figure 7A,B*), despite the reappearance of β_2_ARs-cAMP response back to the T-tubules, as shown previously in.^30^

### 3.7 **β**_2_AR-dependent cAMP signal compartmentation in failing cardiomyocytes is not restored after overexpression of JPH2

To find out if introducing exogenous JHP2 normalizes cell structure and restricts β_2_AR-dependent cAMP response, we transduced failing cells at 16-week post-MI with a virus construct expressing JPH2. Transfection increased JPH2 expression and greatly restored its regular distribution throughout the cardiomyocytes (see supplementary material online, *FigureS3B*); however, this improved neither cell surface topography (see supplementary material online, *FigureS3C*) nor β_2_AR-dependent cAMP signalling (see supplementary material online, *FigureS4*). In cells expressing JPH2, the whole-cell FRET response to β_2_AR stimulation remained low (see supplementary material online, *FigureS4A,B,C)* and the AC-dependent whole-cell cAMP response remained low as well (see supplementary material online, *Figure 4A,B,D*). In addition, reappearance of the β_2_AR-dependent cAMP response back inside the T-tubules did not occur, as seen from the FRET signal changes after local β_2_AR stimulation (see supplementary material online, *Figure 5*). The signal remained diffuse as opposed to the confined signal seen in non-failing myocytes (*Figure 7B*)**.** This data signify that JPH2 alone is not sufficient to confer local restriction of β_2_AR signalling in failing cardiomyocytes.

### 3.8 Changes in G_i_ coupling release the **β**_2_AR-dependent cAMP response from T-tubules confinement

β_2_ARs are known to couple with both G_i_ and G_s_ downstream effector proteins.^8^ Whereas G_s_ stimulates AC and leads to cAMP production, activation of G_i_ is known to inhibit cAMP production.^7^ The latter is involved in restricting cAMP signal to T-tubules in non-failing cells, along with other mechanisms, including phosphodiesterase-dependent cAMP degradation.^13^ To determine if the loss of cAMP confinement in failing cardiomyocytes is due to altered β_2_AR coupling to G_i_, we reanalysed the results obtained previously in.^12^ from non-failing ventricular myocytes treated with PTX. Analysis of cAMP diffusion in these cells showed that G_i_ inhibition indeed led to cAMP production becoming more diffuse, in a manner similar to HF cells (*Figure 7B*). To investigate the coupling of β_2_AR-G_i_ at the onset of HF, we also stimulated cardiomyocytes at 4-week post-MI locally inside T-tubules with and without pre-treatment with PTX (see supplementary material online, *Figure 7*). Higher or lower G_i_ signalling would be reflected in altered β_2_AR-cAMP levels. However, we saw no potentiation of the response of β_2_AR-cAMP by PTX in the T-tubules of cells 4-week post-MI. This indicates that in myocytes 4-week post-MI potential β_2_AR-G_i_ coupling changes are not the reason for the observed diffuse signalling.

## 4. Discussion

**Figure 8:**
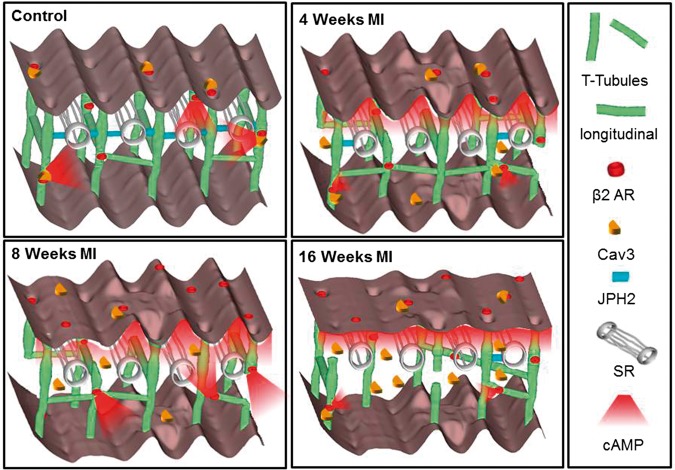
Schematic depiction of changes in structure and location of β2ARs during progression of heart failure. In normal cardiomyocytes the external surface structure (Z-grooves and crests) and internal TAT network, consisting of T-tubules and longitudinal elements, is intact and β_2_AR are located exclusively inside T-tubules; JPH2 connects T-tubules with the SR; cAMP does not diffuse far from the site of β_2_AR activation; and Cav 3 is predominantly in the membrane of cells. In heart failure the surface structure regularity deteriorates progressively; First, after only 4 weeks of MI, JPH2 is downregulated; Second, as early as 4 weeks, the amount of longitudinal elements increases, perhaps as a compensatory mechanism; At the same time β_2_AR-cAMP responses start to appear at the crest, and the cAMP response is no longer confined to the site of β_2_AR activation; Later, at 16 weeks the deterioration of surface and T-tubule structure continues, overall Cav 3 levels increase while the amount of longitudinal element decreases, and the overall cAMP production decreases as well, which may be due to inefficient β_2_AR associated AC signalling.

This study combines evidence of the structural changes in ventricular myocytes with the sequelae of functional remodelling of local β_2_AR-cAMP signalling during progression of LV hypertrophy to chronic HF A summary of these alterations during HF progression can be found as a schematic in *Figure 8*. Rat hearts that underwent experimental MI experienced a progressive remodelling of the remaining viable cardiomyocytes contributing to the progressive decrease in ventricular function. Cardiomyocytes at 4-week post-MI have evidence of early structural and functional changes, confirming that hypertrophic cellular remodelling starts early after MI in the remaining myocardium (see supplementary material online, *Figure 2*). By 8-week post-MI the cardiomyocyte remodelling has progressed more towards the chronic HF phenotype with remodelling of functional β_2_AR-cAMP signalling in addition to structural changes. At 16-week post-MI cardiomyocytes are closer to the end-stage of HF with a severe alteration in all investigated structural features (*Figure 2 and 3*) and the important functional sequalae with reduced β_2_AR-cAMP signalling (*Figure 4*). This classification is corroborated by other groups using the same experimental, rat model where signs of advanced HF were observed after 8-week post-MI e.g. reduction of SERCA2a gene expression,^32^^,^^33^ sympathetic nerve stimulation^3^ and whole heart morphological and functional changes.^34^

### 4.1 Structural changes during HF progression

After cardiac injury, the TAT network undergoes progressive and complex structural remodelling. Initially, this involves the increase of longitudinal elements. Disruption of the highly ordered cardiomyocyte structure, in particular disorganization of the TAT network in the LV, has been observed in a variety of coronary artery occlusion models.^2^ After transverse aortic banding in rat, T-tubular loss of around 10% already appears in compensated hypertrophy.^25^ This T-tubular loss becomes much more severe (up to 86%) at the early onset of HF and at advanced HF.^25^ However, a recent, more detailed study of the T-tubule system after arterial ligation in mice using stimulated emission depletion microscopy reported progressive reorganization and partial elongation of T-tubules after 4 and 8 weeks which is undetectable by conventional confocal microscopy.^26^ In this study, we discovered that the Z-grooves reduced steadily over the 16 weeks in the observation period following MI, with the appearance of smooth areas on the surface and the disappearance of T-tubule openings (*Figure 2*). In contrast, severe disorganization in the regularity and a moderate decrease in the density of the internal TAT network were already evident at 4-week post-MI (*Figure 2*), and persisted until 16-week post-MI. At 4- and 8-week post-MI, an increased amount of longitudinal TAT elements appeared which may reflect myocardial hypertrophy. This early increase in the amount of longitudinal tubules is lost again at 16-week post-MI at the end-stage of HF. Similar changes were previously reported in HF models in different species,^26^^,^^35^^–^^37^ which were associated among other things to changes in Cav-3 levels.^26^

In our study, we observed Cav-3 dissociation early in the remodelling phase post-MI and a late increase of the Cav-3 protein levels through to 16-week post-MI. This predominantly reflected increased Cav-3 protein levels in the cardiomyocyte cytosol. As was shown previously in^26^ Cav-3 levels do not undergo a linear degradation or increase but rather undergo differential changes in HF progression, which are also dependent on the species, model and severity of HF. Wagner *et al.*^26^ reported an increase of Cav-3 at the earlier stages, which they correlated with increased longitudinal elements in the mouse TAT system. In our own rat model of HF we saw no significant alterations in total Cav-3 protein levels at 4- and 8-week post-MI, where we detected elevated numbers of longitudinal elements. However, we detected an increase in Cav-3 at 16-week post-MI, at a time of reduced numbers of longitudinal elements and a severely disrupted TAT system, which contained only T-tubules that were oblique and no longer orderly aligned (see *Figure 3A*). Hence, we assume Cav-3 dissociation to be partially responsible for the reorientation of T-tubules into the longitudinal direction. A recent publication studied HF in rabbit after ∼7.1 months of aortic insufficiency and constriction.^38^ In this rabbit model a Cav-3 protein reduction was shown together with an increased β_2_AR signal in the myofilament compartment of cardiomyocytes and unaltered signalling levels at the sarcoplasmic reticulum (SR) and plasma membrane. In our rat model at 16-week post-MI we observe an overall decrease of β_2_AR signalling, potentially reflecting a more advanced HF phenotype.

HF has been shown to be accompanied by cardiomyocyte dedifferentiation and a reactivation of the fetal gene programme.^39^ Therefore, the structural remodelling observed may also reflect a return to a more ‘fetal phenotype’. Although investigating possible molecular mechanisms underpinning the early structural remodelling, we found that one of the proteins involved in the maintenance of T-tubules, JPH2, decreased dramatically as early as 4 weeks and remained low throughout the progression towards HF (*Figure 2*). JPH2 maintains the junctions between TAT elements and the SR^40^ and its expression are decreased during hypertrophy and HF.^41^ Remodelling of the TAT network hence may in part be triggered by down-regulation of JPH2. It is well established, that primary cells in culture suffer from dedifferentiation.^42^ Especially the FRET experiments described here have to be interpreted with this fact in mind. During the structural investigation of cardiomyocytes after only 1 h of culture in comparison to cardiomyocytes after 48 h of culture we revealed TAT network damage similar to that observed in cells freshly isolated from animals with MI 4-week post-MI (see supplementary material online, *FigureS6*).

### 4.2 **β**2AR-cAMP response appearance at crest accompanies structural remodelling

The integrity of T-tubules is intimately linked to efficient excitation–contraction coupling. Localized cAMP signalling, in particular via β_2_ARs, is also believed to be dependent on an intact TAT network. It is important to note that β_2_ARs can switch from stimulating AC-dependent cAMP production via stimulatory G_s_ proteins to inhibiting it via inhibitory G_i_ proteins. The latter prevents excessive βAR-mediated G_s_ activation and cardiotoxicity by suppressing cAMP production through the deactivation of G-proteins and the internalization of receptors.^43^ If β_2_ARs predominantly exhibit G_i_ coupling, a change in cAMP levels may no longer be detectable but β_2_ARs can still play a role in cell physiology/pathophysiology by regulating kinase-dependent signalling and gene expression pathways.^44^ We have previously shown that in healthy cardiomyocytes, functional β_2_ARs are exclusively situated inside T-tubules.^13^ In end-stage HF, however, functionally detectable β_2_AR redistribute onto surface crests.^13^ In this study, we observed that after whole-cell stimulation of β_2_ARs, cAMP levels were slightly decreased at 4- and 16-weeks post-MI (*Figure 4*). However, they were increased at 8 weeks. The total AC activity stimulated by NKH477 was not significantly affected at the earlier stages of remodelling (4 and 8 weeks) but was significantly reduced at 16-week post-MI. This may signify that changes in global cAMP levels only happen in the end stage of HF. Localized stimulation via SICM nanopipette demonstrated a significant reduction of the β_2_AR-cAMP response at 4 weeks in the T-tubules with no simultaneous increase of the cAMP signal at the crests (*Figure 5*). At 8-week post-MI, local cAMP levels increased in comparison to 4-week post-MI after stimulating at the cell crests, explaining the changes observed in the whole-cell cAMP studies (*Figures 4 and 5*). This may be attributed to a hypertrophic, compensatory response of the myocardium, as well as to the appearance of the β_2_AR receptor response to the crest at 8-week post-MI. An increase in G_i_ protein expression but not activity has been reported in a rat HF model,^45^ which makes us assume that the observed decrease of cAMP production at 16-week post-MI is due to a reduction in AC activity and not due to increased β_2_AR signalling via G_i_. This is consistent with the previous observation that β_2_AR expression remains unchanged during HF.^4^ Our study may explain how spatial and functional β_2_AR alterations can still lead to an advanced HF phenotype. Our study also shows unrestricted cAMP diffusion throughout the cytosol as early as 4-week post-MI (*Figure 6*). The observed changes in cAMP compartmentation could conceivably be a consequence of the extensive loss of the T-tubules and, as we have shown previously, decreased β_2_AR association to the scaffolding protein Cav-3.^23^ Our observations indicate that progressive alterations in signalling initially result from reduced β_2_AR-cAMP production. Consecutively, a combination of functional β_2_AR response appearance at crests and loss of cAMP compartmentation takes place. Alterations of the TAT system and the down regulation of JPH2 precede the appearance of the functional β_2_AR response and potentially affect the confinement of β_2_AR-cAMP. Interestingly, in a model with structural recovery of the TAT network after HF using SERCA2a gene therapy, there was no restoration of JPH2 expression, which was accompanied by persistent diffuse β_2_AR-cAMP signals, even though these signals were again exclusively inducible inside T-tubules. So, perhaps these early changes in cell structure allow spatial rearrangement of secondary messenger pathways and signalling via G_i_ proteins. Hence β_2_AR-cAMP appearance at the crests may compensate for the pronounced loss in β_1_AR activity reported by other groups^45^^,^^46^ but ultimately contributes to generation of HF.

### 4.3 Mechanisms of TAT network and **β**2AR signalling rearrangement

During HF progression, we observed cellular changes which are not yet fully understood mechanistically. In the acute period following infarction cardiomyocytes are exposed to increased wall stress and neuro-hormonal activation. This activates hypertrophic signalling and the development of a hypertrophic phenotype. T-tubular structures are able to respond to load via a stretch sensitive complex.^47^ The MI-induced mechanical overload could trigger a decrease in T-tubule regularity and density,^48^ whereas longitudinal elements have been shown to temporally increase together with Cav-3 protein aggregation.^36^ JPH2, which couples the T-tubules to the SR may also be involved in stretch sensing as its knockout leads to cell hypertrophy.^48^ The protein also co-localizes with ryanodine receptors to strongly increase the efficiency of excitation–contraction coupling,^49^ which is far less efficient in HF.

We have previously demonstrated that the localization of β_2_ARs in non-failing ventricular myocytes depends on cholesterol-^13^ and Cav-3-rich microdomains.^23^ Here we show that the β_2_AR-cAMP responses are relocated beyond the T-tubule membranes around 8-week post-MI, while β_2_ARs-dependent cAMP signalling loses its compartmentation earlier at 4-week post-MI. The latter correlates temporarily with a reduction in JPH2 protein levels, dissociation of Cav-3 into the cytosol and a parallel rise in longitudinal elements, as was reported previously in^26^ and confirmed in our study at 4-week post-MI (*Figure 3A,B*). We have shown previously that Cav-3 is also involved in β_2_AR signal regulation. Failing rat ventricular myocytes overexpressing Cav-3 for 48 h demonstrated redistribution of β_2_AR’s functional effects back into the T-tubules, suggesting that Cav-3 might potentially restore the β_2_AR-mediated cAMP localization and its confined signalling.^23^ In this previous publication^23^ we did not measure Cav-3 protein levels at 16-week post-MI, but observed that Cav-3 overexpression partially restored the β_2_-AR signal back into T-tubules, which we correlated to the increased reformation of caveolae structures. By measuring Cav-3 membrane and cytosol fractions at 4- and 16-week post-MI (*Figure 3E,F,I*), we now understand that Cav-3 starts dissociating into the cytosol in the early phase of remodelling post-MI. Cav-3 remains increased in the cytosol at 16-week post-MI, and reduced sarcolemmal Cav-3 levels may explain why β_2_AR-dependent cAMP signalling levels are significantly decreased at this stage. The observed changes in Cav-3 protein levels may contribute to the progression of HF, because Cav-3, via its scaffolding domain, binds a variety of important signalling molecules, including PKA, via a scaffolding domain to form signalling complexes and regulates their activity.^50^ Activation of PKA by cAMP leads to a local increase in phosphodiesterase 4 activity, which provides a negative-feedback control of the cAMP levels.^13^ Inhibition of phosphodiesterase 4 by rolipram reproduced increased cAMP diffusion in non-failing rat ventricular myocytes.^13^ Therefore, cAMP propagation observed at 4-week post-MI, could be indicative of a loss of efficient cAMP degradation via phosphodiesterases due to Cav-3-dependent disruption of PKA activity and/or localization. In addition, Cav-3 dissociation observed after MI,^51^ may also cause a decrease in JPH2^30^ affecting the bridging between the SR and TAT network as well as dissociation of β_2_AR with their molecular partners within the signalosome. However, the mechanisms underlying functional β_2_AR response redistribution from T-tubules onto crest structures still remain elusive and require further investigation.

### 4.4 Conclusions and outlook

Our study reveals time-dependent alterations in the cardiomyocyte TAT structure and the expression of structural proteins such as JPH-2 and Cav-3, which gradually alter the β_2_AR-cAMP response location as well as β_2_AR-cAMP signalling characteristics. Our observations contribute to understanding of the biphasic remodelling of β-adrenergic signalling following MI during the sub-acute and chronic phases in the progression to HF. The identification of early JPH down-regulation, TAT remodelling and later redistribution of functional β_2_AR signalling, has potential implications for the optimal selection of treatment strategies to prevent the transition from hypertrophy towards HF. We propose that interventions to prevent the structural remodelling of the TAT system early in the disease may maintain the TAT-SR spatial relationship, and potentially sarcolemmal structural remodelling. For example, beta-blockers with different β_1_AR:β_2_AR selectivity may have different actions at progressive stages of HF development.^52^ This could conceivably maintain β_2_AR localization and the native spatial restriction of β_2_AR signalling, and may represent a new potential therapeutic avenue.

## Supplementary material

Supplementary material is available at *Cardiovascular Research* online.

## Supplementary Material

Supplementary Data
