# Supplementary material for: T-tubule remodelling disturbs localized β2-adrenergic signalling in rat ventricular myocytes during the progression of heart failure
Source: Cardiovasc Res. 2017 May 13;113(7):770–82. doi: 10.1093/cvr/cvx074 (PMC5437368; doi:10.1093/cvr/cvx074)
Supplement: Supplementary Data [file cvx074_Supp.zip › Supplementary materials Schobesberger et al2017.docx]

**SUPPLEMENTARY MATERIAL**

**Supplementary Methods**

**Reagents:**

If not indicated otherwise reagents were obtained from Sigma Aldrich, UK.

**Detailed methods:**

**Induction of myocardial infarct to generate a rat heart failure model with progressive cardiac cell remodelling**

For the generation of the rat heart failure model the respective animals (weighing 250g) were anaesthetised with isoflurane (5% reduced to 2% once intubated and ventilated) and were given the antibioticum enrofloxacin (5 mg/kg) and 0.9% saline (10 ml/kg) as well as buprenorphine subcutaneously (0.05 mg/kg) for pain relief. Then both the chest and the pericardium were opened surgically and a suture was bound around the left anterior descending (LAD) artery and tightened to constrict the blood flow. After the ligation the animals were closed up again. At any sign of distress further pain killers were administered^1^.

**Calculation of HF markers**

The ejection fraction volume of control hearts and hearts 4, 8 and 16 weeks post-MI hearts was calculated from M-mode echocardiography recordings (Vevo 770 micro-imaging system, Visualsonics) as a marker of alterations in wall contractile functionality. Additionally, the heart weight to tibia length (HW/TL) ratio was measured after isolation of the respective hearts as an indicator of progressive hypertrophy.

**Cell culture**

Isolated cardiomyocytes were plated onto laminin-coated glass bottom dishes (MatTeK corporation, Ashland, USA) and incubated at 5% CO2 in modified M199 (Invitrogen, UK) culture medium containing per 500 ml bovine serum albumin (0.5 g/L), creatine (5 mmol/L), taurine (5 mmol/L), L-ascorbic acid (100 µmol/L), carnitine (2 mmol/L), and penicillin/streptomycin (100 mmol/L). Before experiments the medium was replaced with a physiological solution (pH7.4) containing NaCl (144 mmol/L), KCl (5 mmol/L), HEPES (10 mmol/L) and MgCl2 (1 mmol/L). All experiments were performed at room temperature (20-22°C).

**Z-groove index calculation:**

The Z-groove index introduced by our group^2^ is a ratio between the observed Z-groove length and the theoretical maximum (extrapolated, maximally possible). It is used to assess the surface integrity of cardiomyocytes and was therefore calculated for control cells and cells at the 3 different time-points after MI induction (for illustration see *Supplementary Figure* 9).

**T-tubule regularity measurement**

Cardiomyocytes were stained with Di-8-ANEPPS (10μmol/L, Biotium) for 1 min. Then whole cell Z-stacks of optical slices at every 0.49 µm were obtained with an inverted confocal microscope (Zeiss LSM-780) to determine the regularity of the T-tubules in the TAT network. The resulting images of the TAT network were analysed using the freeware ImageJ (<http://rsbweb.nih.gov/ij/>, 2013, NIH) by choosing areas in the middle of Z-slices (at least 2 µm distant from either the top slice or bottom slice) and distant from the cell nuclei or cell edges (at least 2 µm apart). 10 areas of 40 µm x 5 µm size from 10 confocal slices per cell were selected and automatically ‘thresholded’ into binarised images using the default threshold from ImageJ and then plotted into waveforms. The quantification of regularity was done via a power peak transformation of these waveforms using a custom-written macro for Matlab (The MathWorks, Inc., Natick, MA, USA) as described before^2^, where “cycles per micron” is the equivalent to 1/(distance between T-tubules) and the power is indicative of the cumulative frequency of each distance (see Supplementary Figure 8 for a descriptive schematic of the T-tubule regularity calculations). The resulting values were averaged per cell. The output for each power calculation is an arbitrary number that we defined as an indicator of how regular T-tubules appear at a physiological distance of ~2 µm, as described previously^3^. 3D reconstructions of the cardiomyocyte TAT network were generated using the freeware Fiji.

**T-tubule density measurements**

Simultaneously to the T-tubule regularity calculations the 40 µm x 5 µm areas chosen from the confocal slices after Di-8-ANEPPS staining, which were binarised to black and white images, were used to determine the T-tubule density by calculating the percentage of black pixels per area. The obtained values were averaged per cell.

**Determination of the amount of longitudinal elements in the TAT system**

The longitudinal elements are less well studied and a far more irregular feature of cardiomyocytes. Longitudinal elements appear perpendicular to T-tubules and connect some of them but not all. The design of an automatic program that will calculate axial elements is not trivial; therefore an algorithm was designed for semi-automatic quantification. Confocal Z-stacks were taken of cardiomyocytes stained with Di-8-ANEPPS at various time points following MI. An area of 40 µm x 10 µm was chosen from a Z-slice, the same area directly below the first was chosen on the slice below. This process was repeated for 10 consecutive confocal slices in the middle of each Z-stack with a 1 µm separation between slices. Then the areas were processed as follows: 1) the overall TAT structure is detected using Canny edge detection; 2) the detected edges are filled and reduced to a 1 pixel width using low-level, morphological operations; 3) transverse tubule candidates are collected by applying adaptive thresholds to the histograms over the one dimension projections of the detected structures; 4) the detection of spatial regularity/repetition of the transverse tubule is used to make the method more robust towards the errors introduced by the quantization in the z-direction; 5) all transverse tubule elements are identified and removed; 6) the remaining axial elements are quantified and verified by eye. The last step had to be performed as real life TAT data proved to be much more curvilinear than the straight elements which were assumed to exist initially, especially in cells at 16 weeks post-MI.

**3D reconstruction of the Transverse Axial Tubule (TAT) system**

Z-stacks of 10 consecutive, confocal slices each (corresponding to 40 µm length, 10 µm and 5 µm thickness in actual size) were transformed into 3D reconstructions of the TAT system in control and at all 3 progressive stages of MI using the free software Fiji (http://fiji.sc/Fiji).

**Förster Resonance Energy Transfer (FRET) measurements of β_2_AR dependent cAMP signaling**

To perform measurements of intracellular cAMP signaling, isolated cells were infected with an adenoviral vector encoding the FRET-based cAMP sensor construct, cEPAC2^4^. Successful transfection was obtained after 48hrs infection with an approximate MOI of 300.

The imaging system built around a Nikon TE2000 microscope was previously described^5^. The recording of the FRET signal was performed using Micro-Manager 1.4 (Vale Lab, University of California, San Francisco <http://www.micro-manager.org/wiki/Micro-Manager>) with custom made plugins^5^. A custom-made, gravity based perfusion system was employed to deliver drugs into the external bath solution. During calibrations, complete replacement of extracellular solution was achieved in less than 5 seconds. To avoid cross-stimulation of β_1_-ARs all FRET experiments were performed with solutions containing CGP20712A (100nmol/L). Whole-cell β_2_AR stimulation was achieved with Isoproterenol (ISO-100nmol/L); and subsequent whole cell adenylate cyclase (AC) stimulation was elicited by NKH477 (5µmol/L). Data obtained in FRET experiments were corrected for bleed-through of CFP emission into the YFP channel and then normalised to the untreated baseline of each recording as previously described^6^.

**Combined FRET/SICM measurements of local β_2_AR dependent cAMP signaling**

Topography images of 10 µm x 10 µm areas of the respective cells were obtained using SICM with nano-pipettes of ~ 100 MΩ resistance to elucidate surface structures. After blocking ß_1_ARs through the superfusion of CGP20712A (100nmol/L), β_2_ARs were stimulated locally via the SICM nano-pipette containing Isoproterenol (10 µmol/L) and CGP20712A (50 µmol/L) by applying pressure as previously described^4^. The drugs were applied either to the T-tubule openings or to the crest areas, the coordinates of which were determined by observing SICM topography images. The FRET signal was recorded using a Hamamatsu ORCA ER camera (Hamamatsu) and Micro-Manager 1.4 with custom-written FRET analysis plugins as described previously. The propagation of the cAMP response due to β_2_ARs stimulation was studied by measuring the FRET response amplitude at different distances from the original nano-pipette stimulation site. The response was deemed as being local when the amplitude of cAMP dropped by more than 50% when measured at a distance of 30 microns from the pipette. Otherwise the response was considered to be diffusing entirely throughout the cardiomyocyte cytosol.

**JPH2 overexpression and FRET measurements of β_2_AR dependent cAMP signal**

To investigate if we could reintroduce cAMP confinement by reinstating JPH2 expression we performed additional experiments. We transfected cells at 16 weeks post-MI with an adenoviral JPH2 overexpression virus (purchased from Vector Biolabs #ADV-212592) for 48h in culture. We then performed immunocytochemical staining against JPH2 to see if levels had increased (see supplementary figure 2) and conducted whole cell (see supplementary figure 3) and local (supplementary figure 4) cAMP level measurements. However, JPH2 overexpression in culture lead to no significant changes in overall cAMP level amplitudes or cAMP diffusion confinement.

**Cardiomyocyte fractionation and Caveolin 3 detection**

Cells were snap frozen and kept at -80 degrees before being lysed by thawing them on ice and incubating them in 200µl of Tris-buffer pH 7.4, 1% Triton with 1 tablette of PhospoSTOP Easypack (Roche, #04906837001) and protease inhibitor cOmplete^TM^ (Roche, # 04693116001)/10ml on ice for 15 min and trituration (x12) with Omnican®100 insulin needles 30g 1/2" (Braun Medical Ltd., # 9151133). Cell lysates were centrifuged in a table top centrifuge at 13.300 rpm and 4°C for 20 min. The supernatant (cytosolic fraction) was transferred into a new, pre-cooled tube, whereas the pellet (membrane fraction) was resuspended in a further 200µl of Tris-buffer pH 7.3, 0.1% Tween with 1 tablette of PhospoSTOP Easypack (Roche, #04906837001) and protease inhibitor Complete (Roche)/10ml. The protein content of both fractions was determined by using Pierce BCA protein kit (Thermo Scientific, #23225) before normalisation via dilution in fractionation buffer of the membrane and cytosolic fractions to each other and resuspension in the respective amount of 3x Laemmli buffer containing 1M Tris-Cl pH 6.8, 20%SDS, 30% Glycerol, 15% β-mercaptoethanol and 0,03% bromphenol blue. Approximately 20µg of the normalised protein fractions were loaded onto and run on a 12% electrophoresis gel and transferred onto PFDV membrane via wet transfer before Ponceau staining for 5 min followed by washing with ddH2O and blocking in 5% low fat milk or 5% BSA respectively and being stained against Cav-3 monoclonal mouse (1:5000), Santa Cruz, sc-5310, diluted 1:5000 in 5% low fat milk O/N at 4°C or SERCA2a monoclonal mouse (1:600), ENZO, ALX-804-088-R100 in 5% BSA to mark membrane fractions and GAPDH monoclonal mouse (1:160000), Biotrend, 5G4, in 5% milk to mark cytosol fractions and with secondary goat anti mouse antibody (1:5000), Bio-RAD, 170-5047, diluted in 5% milk or 5% BSA for 1h before being developed on film. Intermediary washing steps before the secondary antibody and before development on film were 3 x 15 min in TBS buffer pH 7.5 with 0,1% Tween.

**Total protein detection via Western blotting**

Total protein was obtained by lysing cells in RIPA buffer containing 150 mM NaCl, 5mM EDTA pH 8.0, 50 mM Tris, pH 8.0, 1% NP-40 (IGEPAL #630), 0.5% sodium deoxycholate, 0.1% SDS, 1 PhospoSTOP Easypack (Roche, #04906837001) and 1 protease inhibitor cOmplete^TM^ tablette (Roche, # 04693116001)/10 ml and trituration (x12) with Omnican®100 insulin needles 30g 1/2" (Braun Medical Ltd., # 9151133) plus 1x Protease/Phosphatase Inhibitor Cocktail and determining the total protein with a Pierce BCA protein kit (Thermo Scientific, #23225) before adding the respective amounts of 3xLaemmli buffer containing 1M Tris-Cl pH 6.8, 20%SDS, 30% Glycerol, 15% β-mercaptoethanol and 0,03% bromphenol blue. Samples were then loaded on 12% Gels and respective proteins were detected using specific antibodies: JPH-2 goat polyclonal (1:100), Santa Cruz, sc-51313; Cav-3 monoclonal mouse (1:5000), Santa Cruz, sc-5310 and normalisation to GAPDH (1:1000) (mouse monoclonal, BioTrend Chemikalien GmbH, #5G4-6C5) and CSQ (1:5000) (rabbit polyclonal, Thermo Scientific, PA1-913). For each total protein blot approximately 40µg of the protein were loaded onto and run on a 12% electrophoresis gel and transferred onto PFDV membrane before ponceau staining for 5 min followed by blocking in 5% low fat milk (for Cav3, CSQ and JPH-2) O/N at 4°C and with secondary goat anti mouse antibody (1:5000) (for Cav3), Bio-RAD, 170-5047, diluted in 5% milk or with secondary goat anti-rabbit in 5% milk (for JPH-2 and CSQ) for 1h before being developed on film. Intermediary washing steps before the secondary antibody and before development on film were 3 x 15 min in TBS buffer pH 7.5 with 0,1% Tween.

**Statistical analysis software**

The data was statistically analyzed using OriginPro 8.6 (OriginLab Corporation) and GraphPad Prism 5 (GraphPad Software Inc.).

**Supplementary References**

(1) Gorelik, J., Yang, L. Q., Zhang, Y., Lab, M., Korchev, Y., & Harding, S. E. A novel Z-groove index characterizing myocardial surface structure. *Cardiovasc. Res*. 2006;**72**, 422-429.

(2) Lyon AR, MacLeod KT, Zhang YF, Garcia EF, Kanda GK, Lab, M, Korchev YE, Harding SE, Gorelik J. Loss of T-tubules and other changes to surface topography in ventricular myocytes from failing human and rat heart. *Proc Natl Acad Sci USA*. 2009; **106**:6854-9

(3) Ibrahim M, Al Masri A, Navaratnarajah M, Siedlecka U, Soppa GK, Moshkov A, Al-Saud SA, Gorelik J, Yacoub MH, Terracciano CM. Prolonged mechanical unloading affects cardiomyocyteexcitation-contraction coupling, transverse-tubule structure, and the cell surface. *FASEB J*. 2010;**24**:3321-3329.

(4) Wei S, Guo A, Chen B, Kutschke W, Xie YP, Zimmerman K, Weiss RM, Anderson ME, Cheng H, Song LS. T-tubule remodeling during transition from hypertrophy to heart failure. *Circ Res* 2010;**107**:520-531.

(5) Nikolaev VO, Bunemann M, Hein L, Hannawacker A, Lohse MJ. Novel single chain cAMP sensors for receptor-induced signal propagation. *J Biol Chem*. 2004; **279**:37215-37218.

(6) Nikolaev VO, Moshkov AF, Lyon AR, Miragoli M, Novak P, Paur H, Lohse MJ, Korchev YE, Harding SE, Gorelik J. Beta2-adrenergic receptor redistribution in heart failure changes cAMP compartmentation. *Science*. 2010; **327**:1653-7


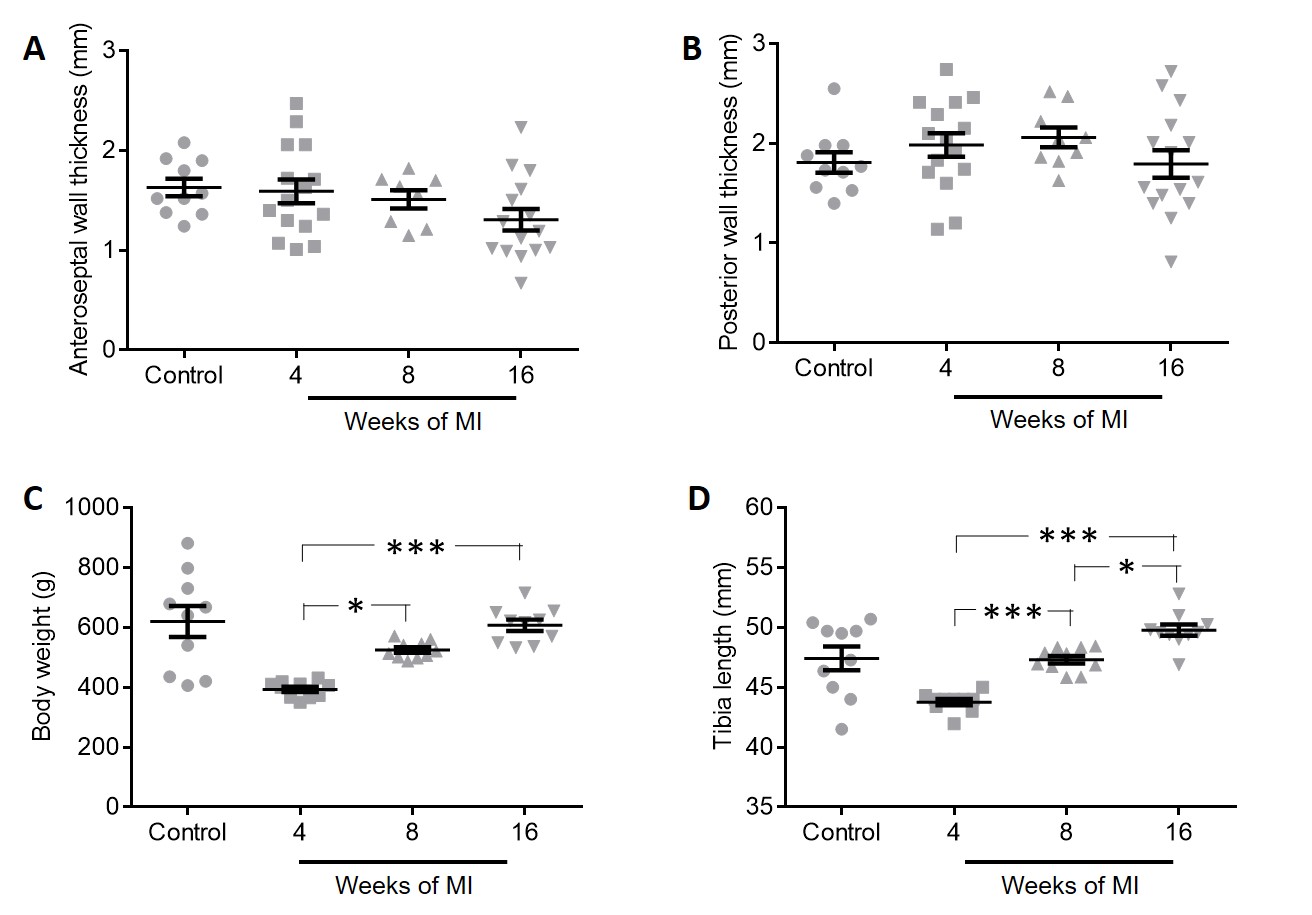


**Supplementary Figure 1. Anteroseptal and posterior wall thickness do not alter while body weight and tibia length increase with the age of the animals.** Anteroseptal and posterior wall thickness was determined throughout the progression of heart failure together with animal body weight and tibia length, control and chronological MI sample numbers are N=10, N=15, N=8, N=15 for anteroseptal wall thickness, N=10, N=15, N=9, N=15 for posterior wall thickness and N=10 each for body weight and tibia length. Control rats were from an even spread of time points 4 at 4 weeks, 4 at 8 weeks and 3 at 16 weeks. N=number of animals. *p<0.05,***p<0.0001 as determined by 1-way ANOVA followed by Bonferroni post-hoc correction.


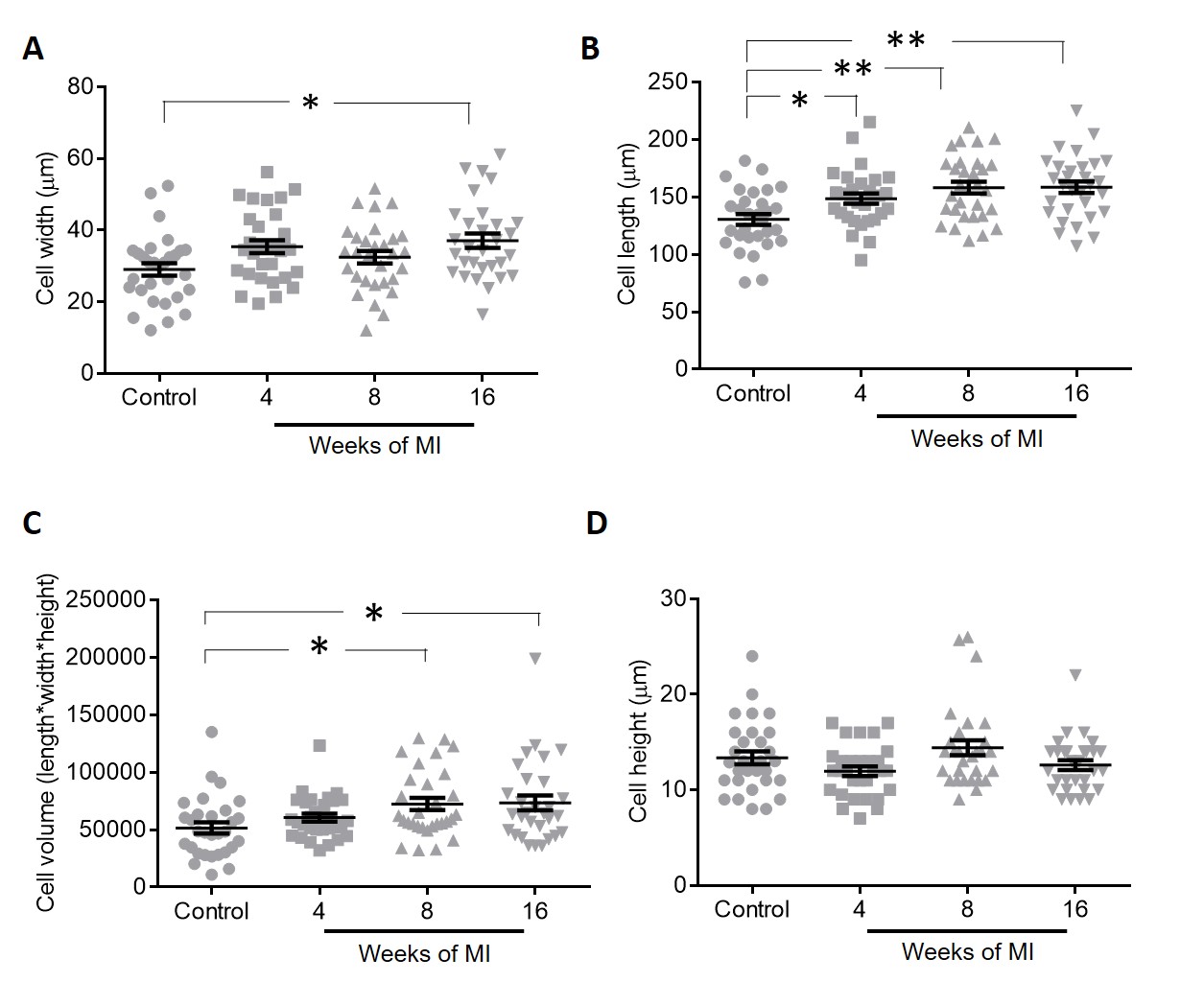


Supplementary Figure 2. Left ventricular cardiomyocyte cell size and volume measurements of single cardiomyocytes isolated from MI rat model. Cardiomyocyte (A) Width (B) length and (C) height were obtained from confocal Z-stack images after Di-8-ANEPPS staining and (E) volume was calculated from these values by multiplication. N/n=4/20 each, N=number of animals/n=numbers of cells. *p<0.05,**p<0.001 as determined via mixed ANOVA followed by Wald χ2-test.


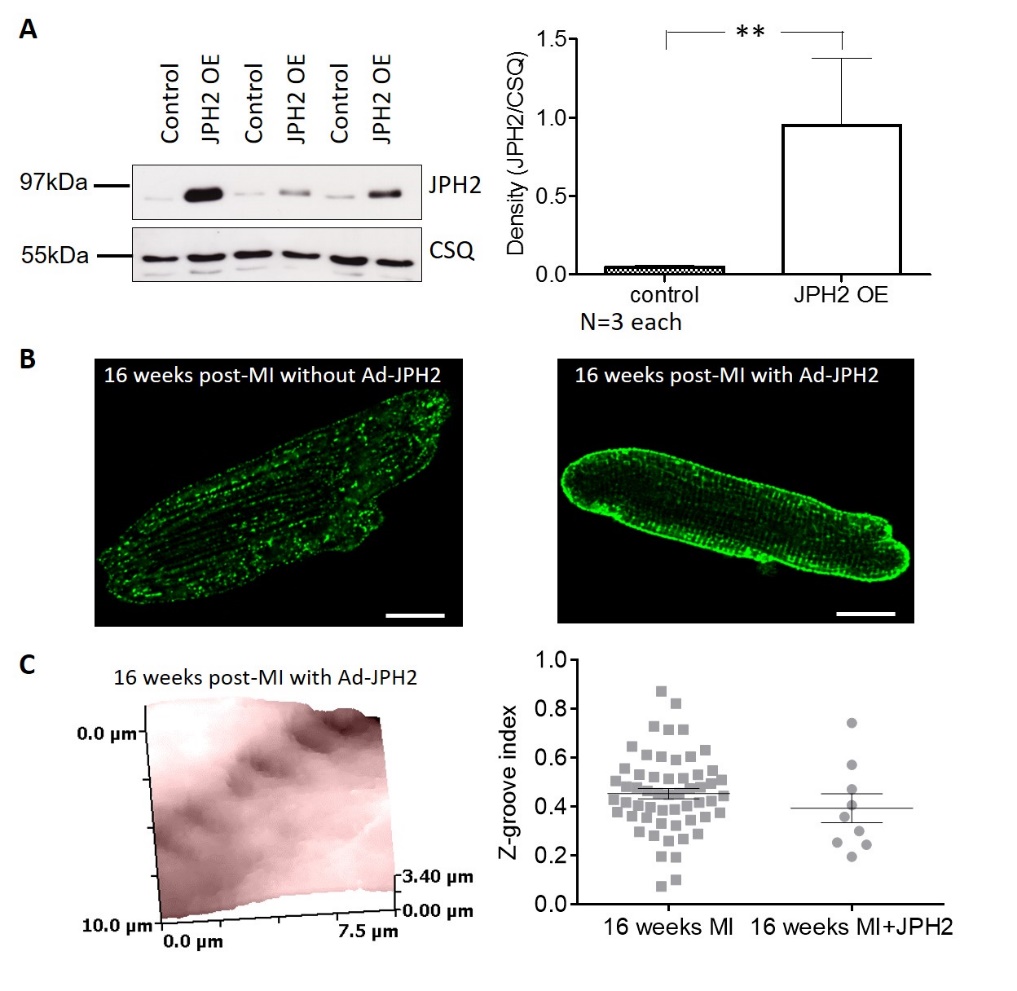


**Supplementary Figure 3. JPH2 virus increases JPH2 expression, without influencing surface structures of cardiomyocytes 16 weeks post-MI. (A)** Western blot detection of JPH2 levels in control cardiomyocytes and cardiomyocytes from the same animals with adenoviral overexpression of JPH2 after 48h of culture. JPH2 levels increase approximately 10 times to the endogenous JPH2 levels. **p<0.001 as determined via two-sided T-test. **(B)** Cardiomyocytes at 16 weeks post-MI were transfected with an adenoviral JPH2 overexpression virus (Ad-JPH2). The transfection efficiency was ~98%**.** Immunocytochemical staining of Junctophilin 2 (JPH2) in cardiomyocytes 16 weeks post-MI without and with JPH2 overexpression, N=3 each.; Scale bar = 20μm. **(C)** Representative SICM surface scan of a cardiomyocyte 16 weeks post-MI transduced with a JPH2 overexpression virus. Z-groove index of cardiomyocytes at 16 weeks post-MI with (N/n=2/9) and without (N/n=9/56) JPH2 overexpression for 48h in culture. N=number of animals/n=numbers of cells. Statistically significant differences were calculated with two sided T-test, but no difference was detected.


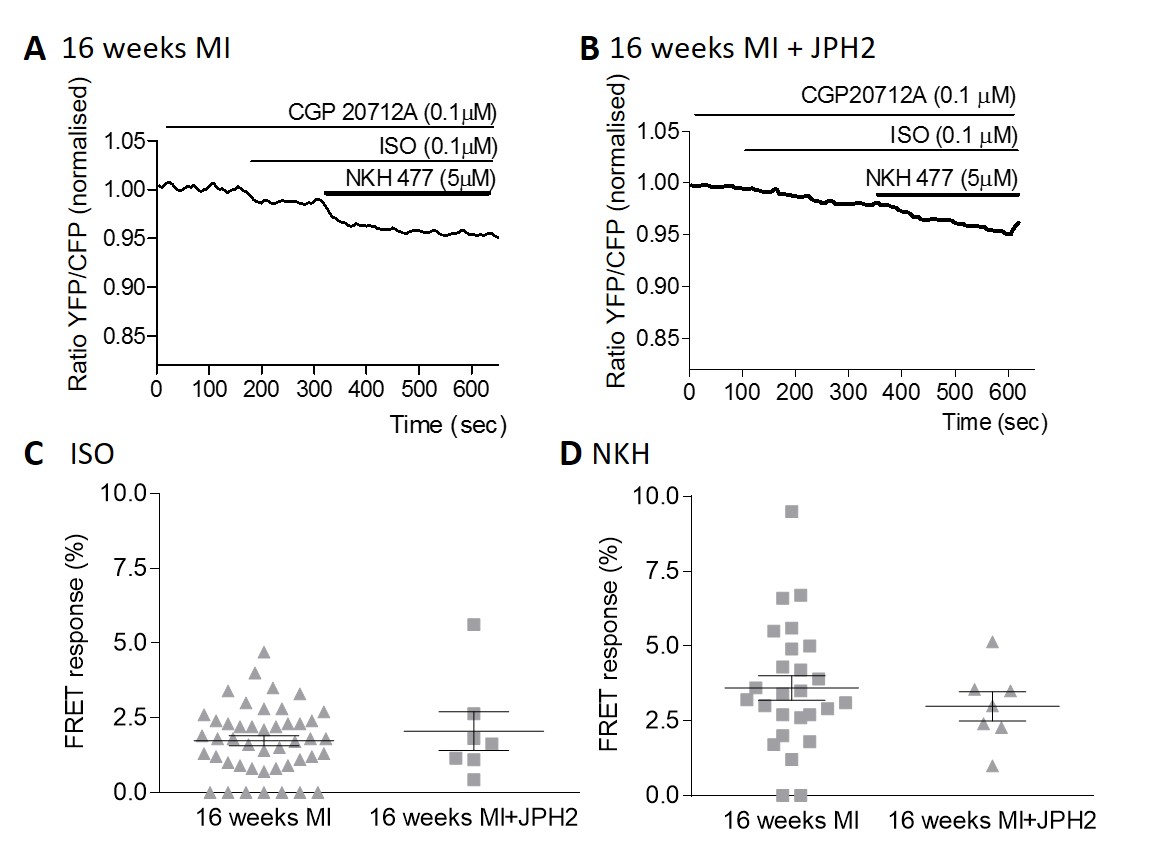


**Supplementary Figure 4**. **JPH2 does not improve either β_2_AR-dependent cAMP response or overall cAMP production. (A,B)** From left to right: Representative whole cell FRET traces after specific β_2_AR and total adenylate cyclase (AC) stimulation of cardiomyocytes 16 weeks post-MI expressing the cAMP FRET sensor Epac2-camps with and without JPH2 overexpression for 48h in culture. **(C,D)** Quantified β_2_AR-dependent and AC-dependent whole-cell cAMP response measured by FRET in cardiomyocytes 16 weeks post-MI (untransduced cell data is the same as in figure 4 in the main manuscript) expressing the cAMP FRET sensor Epac2-camps and with (N/n=2/7) and without (N/n=7/38) JPH2 overexpression for 48h in culture. N=number of animals/n=numbers of cells. Statistically significant differences were calculated with Mann-Whitney U-test, but no difference was detected.


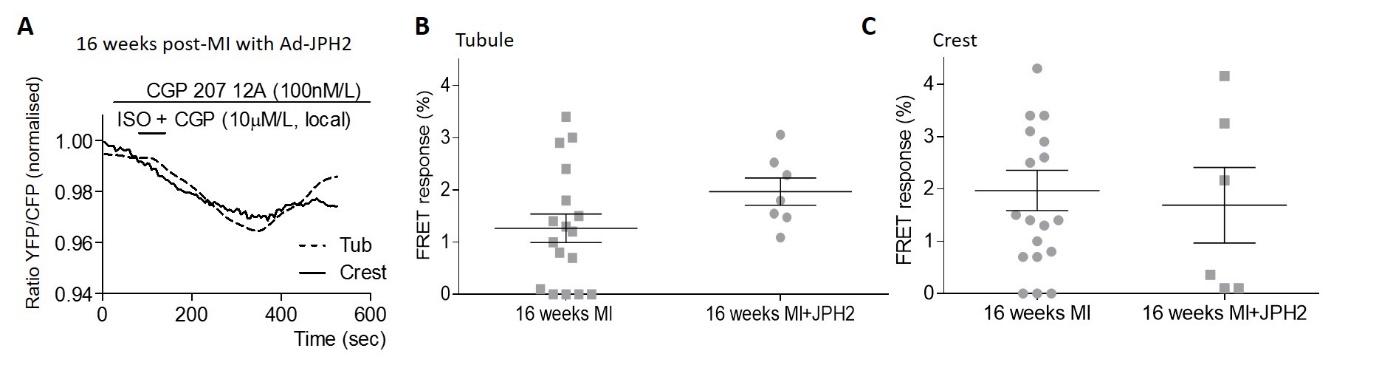


**Supplementary Figure 5**. **JPH2 does not improve local β_2_AR-dependent cAMP response on the crests or on the T-tubules in isolated cardiomyocytes. (A)** Representative traces of β_2_AR-dependent cAMP FRET after local stimulation (with a nanopipette) into T-tubule (dotted line) or crest (black line) in a cardiomyocyte overexpressing JPH2. **(B)** Quantification of β_2_AR-dependent cAMP response to local stimulation in cardiomyocytes 16 weeks post-MI with (in tubules N/n=2/7, in crest N/n=2/6) and without (in tubules N/n=5/17, in crest N/n=6/18) JPH2 overexpression in T-tubules or crests. N=number of animals/n=numbers of cells. No significant differences between the untreated failing and JPH2 overexpressing failing cells was detectable.


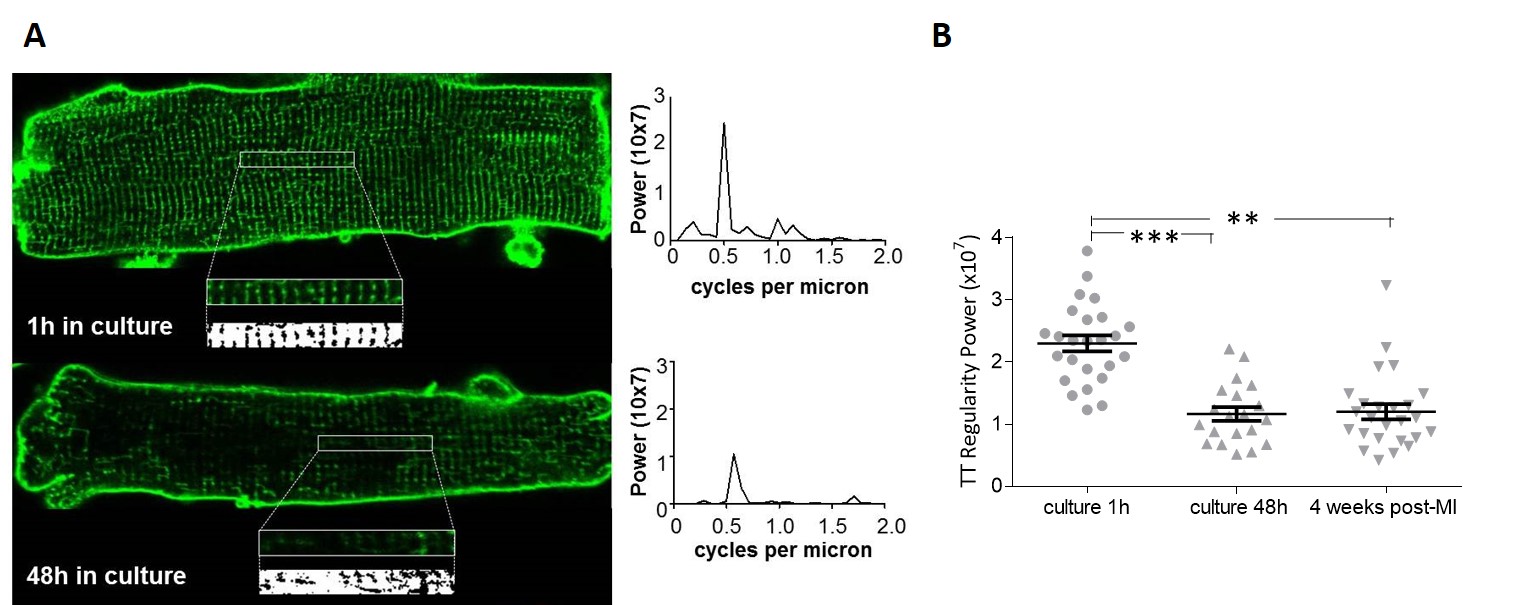


**Supplementary Figure 6 Cardiomyocytes in culture for 48h experience extensive loss in structural integrity.** Cardiomyocytes after 1h and 48h as well as after 4 weeks post-MI (same data as in manuscript Figure 2D) were stained with the lipophilic dye Di-8-ANEPPS and visualised via confocal imaging. N/n=5/25 for 1h in culture, N/n=4/20 for 48h in culture, N/n=5/25 for cardiomyocytes 4 weeks post-MI. N=number of animals/n=numbers of cells. The power of T-Tubule regularity was calculated and compared for statistically significant differences using a 1-way ANOVA; **p<0,001 ***p<0,0001

**
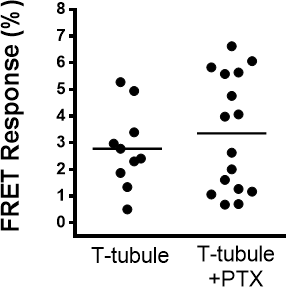
**

**Supplementary Figure 7: Pertussis toxin (PTX) does not potentiate β2AR-cAMP signalling in the t-tubules at 4-weeks post-MI.** Cells at 4 weeks were stimulated locally with ISO in the presence of CGP20712A (with or without Gi removal by PTX treatment). Sample numbers are as follows: untreated tubule N/n = 5/10; PTX tubule N/n=6/16. N= number of animals and n = number of cells. Data not significant, tested with a two-tailed t-test.


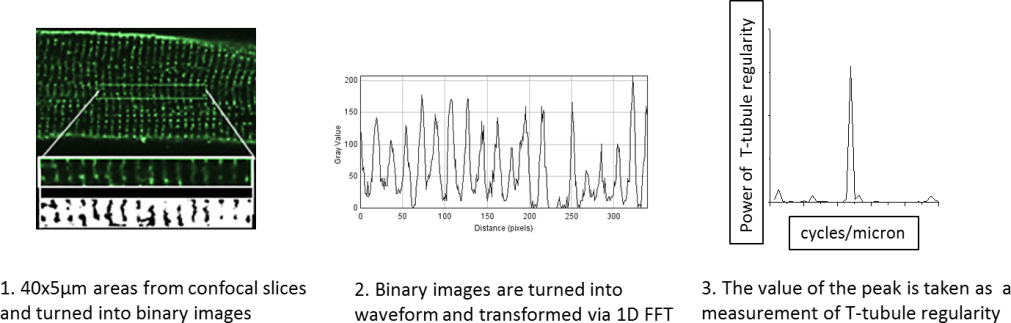


**Supplementary Figure 8. Schematic of T-tubule regularity calculation.** Cardiomyocytes are stained with the lipophilic dye Di-8-ANEPPS and TAT structures are visualised via confocal z-stack imaging. The resulting images are opened up in Image J software and sections correlating to 40x5 µm size are chosen away from the cell membrane or nucelii and binarised. The binarised image sections are turned into wave-forms which undergo 1D Fast Fourier Transformation (1D FFT), resulting in peaks correlating to the presence and regularity of recurring structures. In this case the recurring structures are the transverse oriented tubules of the TAT-network structure.


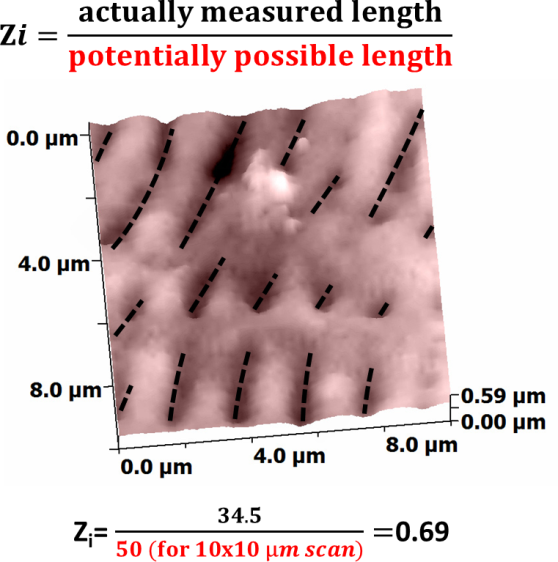


**Supplementary Figure 9. Schematic of Z-groove index determination.** Live cardiomyocyte surface structures are visualised using Scanning Ion Conductance Microscopy. From the resulting scans the Z-groove index is calculated by measuring the length of the Z-grooves present on the scanned cell surface and by dividing the resulting number through the potentially possible length of Z-grooves. Given that Z-grooves are an approximate distance of 2 microns to each other a 10x10 micron surface scan can potentially have a total Z-groove length of 50. The measured length on the above depicted scan amounts to 34.5 microns. Therefore the resulting Z-groove index in this case is 0.69.


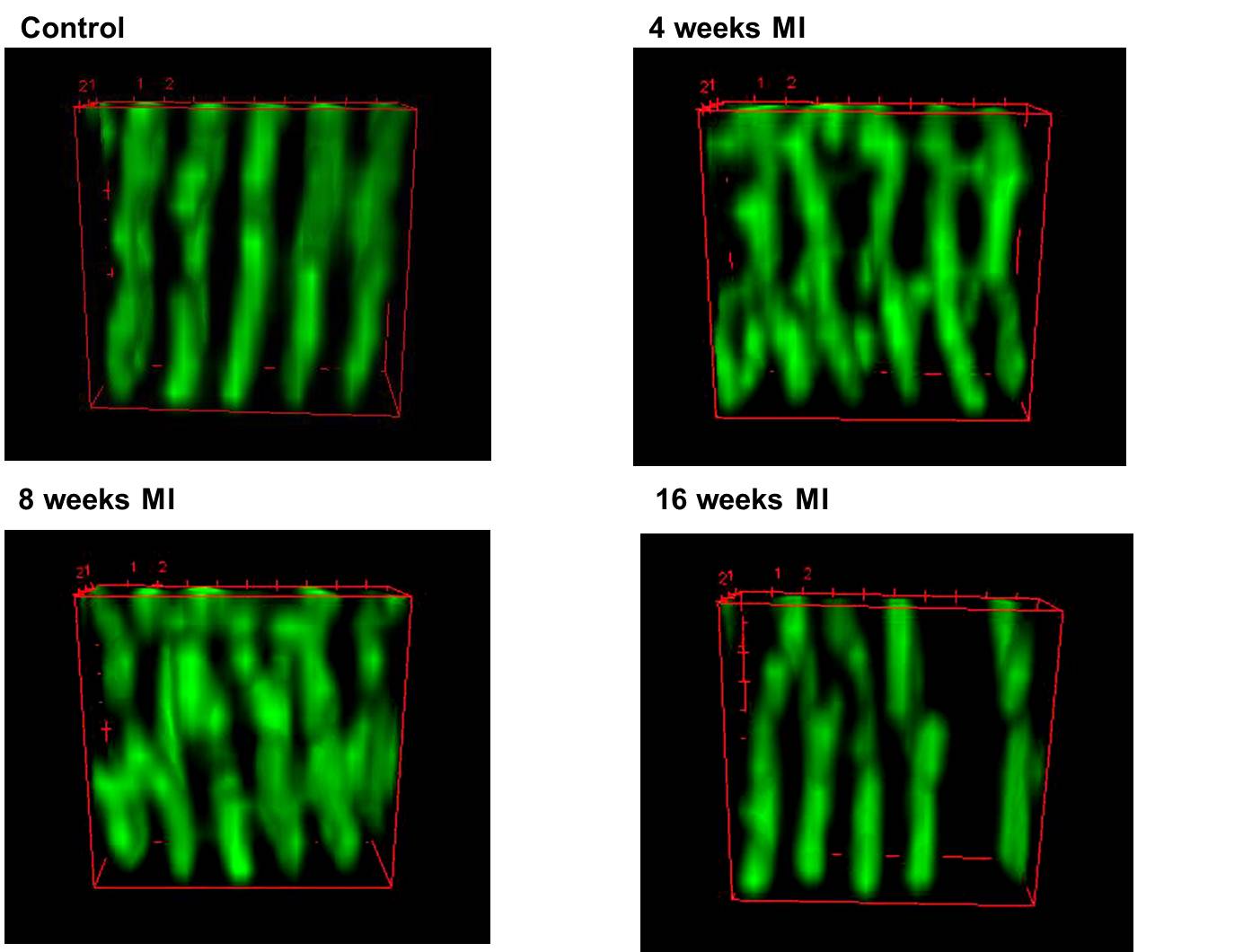


Supplementary Movies. Representations 3D reconstructions of the Transverse Axial Tubule network (example videos) at different time points after MI.
